# Approaches in Sustainable, Biobased Multilayer Packaging Solutions

**DOI:** 10.3390/polym15051184

**Published:** 2023-02-26

**Authors:** Kristina Eissenberger, Arantxa Ballesteros, Robbe De Bisschop, Elodie Bugnicourt, Patrizia Cinelli, Marc Defoin, Elke Demeyer, Siegfried Fürtauer, Claudio Gioia, Lola Gómez, Ramona Hornberger, Constance Ißbrücker, Mara Mennella, Hasso von Pogrell, Laura Rodriguez-Turienzo, Angela Romano, Antonella Rosato, Nadja Saile, Christian Schulz, Katrin Schwede, Laura Sisti, Daniele Spinelli, Max Sturm, Willem Uyttendaele, Steven Verstichel, Markus Schmid

**Affiliations:** 1 Sustainable Packaging Institute SPI, Faculty of Life Sciences, Albstadt-Sigmaringen University, Anton-Günther-Str. 51, 72488 Sigmaringen, Germany; 2 Centro Tecnológico ITENE, Parque Tecnológico, Carrer d’Albert Einstein 1, 46980 Paterna, Spain; 3 Centexbel, Textile Competence Centre, Etienne Sabbelaan 49, 8500 Kortrijk, Belgium; 4 Graphic Packaging International, Fountain Plaza, Belgicastraat 7, 1930 Zaventem, Belgium; 5 Planet Bioplastics S.r.l., Via San Giovanni Bosco 23, 56127 Pisa, Italy; 6 Bostik SA, 420 rue d’Estienne d’Orves, 92700 Colombes, France; 7 Fraunhofer Institute for Process Engineering and Packaging, Materials Development, Giggenhauser Str. 35, 85354 Freising, Germany; 8 Department of Civil, Chemical, Environmental and Materials Engineering, University of Bologna, Via Terracini 28, 40131 Bologna, Italy; 9 AIMPLAS, Plastics Technology Center, Valencia Parc Tecnologic, Carrer de Gustave Eiffel 4, 46980 Paterna, Spain; 10 European Bioplastics e.V. (EUBP), Marienstr. 19/20, 10117 Berlin, Germany; 11 KNEIA S.L., Carrer d’Aribau 168-170, 08036 Barcelona, Spain; 12 IRIS Technology Solutions S.L., Ctra. d’Esplugues 39-41, 08940 Cornellà, Spain; 13 Next Technology Tecnotessile, Chemical Division, Via del Gelso 13, 59100 Prato, Italy; 14 OWS, Dok-Noord 5, 9000 Gent, Belgium

**Keywords:** biobased, multilayer, secondary raw material, coating, end-of-life, sustainability

## Abstract

The depletion of fossil resources and the growing demand for plastic waste reduction has put industries and academic researchers under pressure to develop increasingly sustainable packaging solutions that are both functional and circularly designed. In this review, we provide an overview of the fundamentals and recent advances in biobased packaging materials, including new materials and techniques for their modification as well as their end-of-life scenarios. We also discuss the composition and modification of biobased films and multilayer structures, with particular attention to readily available drop-in solutions, as well as coating techniques. Moreover, we discuss end-of-life factors, including sorting systems, detection methods, composting options, and recycling and upcycling possibilities. Finally, regulatory aspects are pointed out for each application scenario and end-of-life option. Moreover, we discuss the human factor in terms of consumer perception and acceptance of upcycling.

## 1. Introduction

Despite the overall growth in the worldwide production of plastics, European plastic production in 2020 saw a slight decrease from 58 million tonnes in 2019 to 55 million tonnes in 2020 due to the coronavirus pandemic, but is expected to show an even stronger recovery forecasted at +8.5% by the end of 2021, due to global economic upswing (PlasticsEurope, 2021). Currently, the share of bioplastics in the global plastics market remains low at between 1 and 2% of overall plastics production. Yet, the global production capacities of bioplastics are reported to have had a steady growth from 1.9 million tonnes in 2019 to 2.1 million tonnes in 2020 and 2.4 million tonnes in 2021. By 2026, the global production of bioplastics is even forecasted to triple (more than a 200% growth rate in the next five years) and pass the 2% mark for its share of global plastics production [1]. This is an important trend considering the depletion of fossil resources that are needed for the production of conventional non-biobased plastics, which requires more sustainable alternative solutions. Being the largest market for plastics, the packaging sector particularly requires improved circularity from origin to subsequent life cycles as well as increases in bioplastics and other renewably sourced materials in order to contribute to a more responsible use of resources.

In fact, packaging manufacturers are committed to sustainability in terms of reducing the use of raw materials, focusing on the use of materials derived from renewable sources, and re-designing packaging, leading to (a) lower amounts of used packaging material (e.g., lightweighting) and (b) enhanced recyclability, subsequently contributing to and facilitating the circular economy [2,3]. By using multilayer packaging, the reduction in packaging material is accompanied by enhanced barrier properties, since this approach enables the product contained inside to be protected by several layers that are able to provide distinct barrier properties.

Although the approaches mentioned have potential, they are currently limited and not capable of producing a packaging material that is fully sustainable and meets the requirements of a circular bioeconomy. Multi-layered plastic packaging material, for example, is oftenchallenging to recycle. Thus, this kind of packaging material needs to be incinerated or even landfilled [4]. In case of coated fibre-based packaging, depending on the selection of the barrier material, the impact on recyclability may be limited, benefitting from the already high recycling rate for this category of packaging. Therefore, innovative approaches towards a circular bioeconomy and sustainability are needed, and the demand for materials derived from renewable sources is rising.

Bioplastics are plastics that are biobased, biodegradable, or both. They can be used as the main packaging matrix material or as a coating for different substrates, including paper or cardboard, that can also contribute to fully biobased packaging concepts. Bioplastics exhibit numerous advantages, such as replacing fossil-based plastics, renewable feedstocks, and an increased end-of-life (EoL) modularity. Moreover, recent trends in consumer behaviour aiming for waste reduction, greener packaging, and greater sustainability also increase the demand for bioplastics [5]. The human factor, i.e., consumer preferences, is decisive for establishing new and sustainable packaging strategies, and, therefore, it must not be neglected. Innovative packaging solutions suitable for improving sustainability aspects have to not only provide a lower carbon footprint and sustainable EoL options but also maintain barrier properties and subsequently the shelf life of the products contained in order to achieve consumer acceptance [6].

The objective of this review is to present the most recent state of the art of biobased packaging, including their modification strategies and potential applications. In order to provide an expedient and comprehensive overview, this review article focuses on polylactic acid (PLA), polyhydroxyalkanoates (PHA), biobased polyethylene (bioPE), biobased polyethylene terephthalate (bioPET), and fibre-based packaging materials. These materials are the main focus of this research study as it was carried out within the PRESERVE Project: “High performance sustainable bio-based packaging with tailored end of life and upcycled secondary use”, funded by the European Union (EU) Horizon 2020 Research and Innovation Programme under Grant Agreement No. 952983. In order to comprehensively evaluate the sustainability and circularity of these biobased packaging materials, this review outlines and discusses assessment of different EoL options, such as home composting and industrial composting as well as material recycling and upcycling. Examples of recent advances in the development of biobased packaging materials for specific applications are also covered.

## 2. Types of Biobased Packaging Material

According to the European standard EN 16575 (“Biobased Products—Vocabulary”), the term “biobased” means that a material or product is wholly or partly derived from biomass, which can be sourced from carbohydrate-rich plants, such as corn or sugarcane, from ligno-cellulosic feedstock, or from waste. Whereas “biobased” only refers to the type of feedstock source and origin, the term “biodegradable” refers to the EoL of a material. Biodegradation is a biochemical process during which microorganisms that are present in the environment metabolise materials into water, carbon dioxide, and biomass, and artificial additives facilitating the biodegradation process are not needed [7]. The biodegradation process is highly influenced by various environmental factors, as for example temperature, humidity, inoculum, the material to be biodegraded and the specific application may affect the biodegradation rate. Therefore, especially for certification purposes, the term “biodegradability” should only be used when the specific environmental conditions and time frame in which biodegradation can occur are clearly specified, in order to avoid ambiguity.

As both terms describe a different part of the lifecycle of a material, “biobased” does not equal “biodegradable” and vice versa. Therefore, bioplastic packaging materials can be assigned to one of three groups: they can be either biobased, or biodegradable, or both.

**Figure 1:**
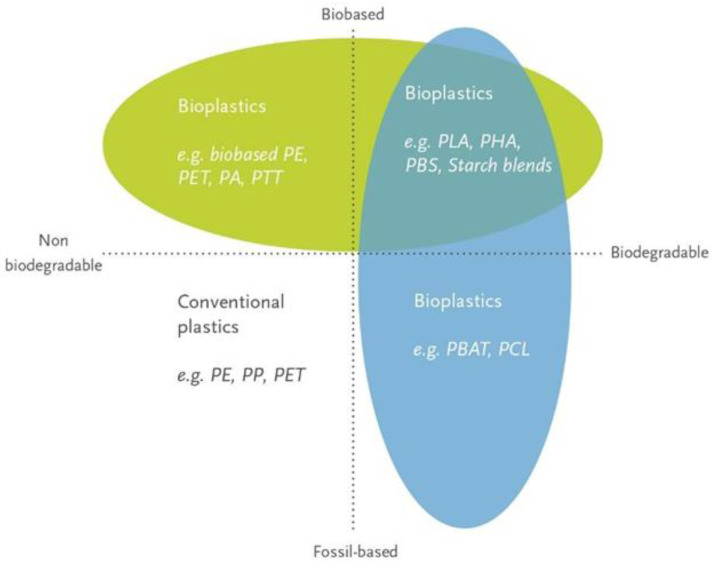
Material coordinate system of bioplastics [8].

Specifically looking at PLA, PHA, bioPE, and bioPET, they are defined by their source of feedstock and possible EoL options. Referring to Figure 1, PLA and PHA are biobased and biodegradable plastics and can be located in the upper right corner of the matrix, whereas bioPE and bioPET can be located in the upper left corner as biobased but non-biodegradable polyolefins or polyester, respectively. The latter are commodity plastics with the same properties as their fossil-based counterparts (conventional PE and PET) but are made from renewable resources; hence, they are also called drop-in solutions (See Section 2.3 and Section 2.4).

As summarized by European Bioplastics (2016) [9] and Rosenboom et al. (2022) [10], different international standards and labels exist for determining and identifying the biobased content of a certain product or sample. These different labels are awarded by different certification institutes. Consequently, the precise certification strategies may differ, but in any case, they rely on accredited international standards. The same holds true for biodegradability certification and labelling. Different certification schemes and corresponding labels are accredited for the European market. For the certification and labelling of biodegradability, however, it needs to first be specified in which environment and according to which standard a material is biodegradable. The requirements for the biodegradability of packaging material in different possible environments, such as industrial composting plants, home and garden composting, marine environments, and soil, are defined for the corresponding standard, if available. More detailed information on certification and labelling are provided by European Bioplastics (2016) [9] and Rosenboom et al. (2022) [10].

Paper and cardboard can also be used to develop high-performance biobased packaging. Fibre in paper and cardboard is generally biodegradable by nature [11]. Nevertheless, in some cases, there is a maximum thickness at which it actually reaches the maximum time period allowed by specific industrial and/or home composting standards. Likewise, the selection of the appropriate additives and coating to meet the requirements set in the respective standards is important.

### 2.1. Polylactic Acid (PLA)

Polylactic acid (PLA) is a versatile material that is already being used successfully in various different packaging applications. It is currently the biobased plastic material with the highest market share, due to its versatility and processability [12]. PLA is synthesised by either the ring-opening polymerisation of lactide (made from lactic acid (LA)) or a direct condensation reaction of LA. Lactic acid can be obtained by the fermentation of glucose, which is derived from hydrolysed corn starch from corn, or sucrose, which is derived from sugarcane. [13] The properties of PLA can vary by the ratio of the stereoisomers used in the synthesis, i.e., D- or L-LA, and by molecular weight. PLA made from either isomer is crystalline. However, high crystallinity increases the brittleness of the material. Most PLA used for packaging applications is poly(L-lactic acid) (PLLA) modified with D-isomer to less than 15% to maintain the necessary crystallinity [14]. PLA has a good oxygen barrier but a poor water vapour barrier and shows high transparency [15]. Typical applications made with PLA currently on the market are mainly rigid packaging, including trays, blisters, containers for fresh produce, or bottles for non-carbonated drinks. PLA is industrial compostable in line with EN 13432 but shows more or less no degradation behaviour in environments with lower temperatures [16]. PLA can also be easily mechanically recycled, and recycled PLA (r-PLA) from post-industrial waste could already be used in the market [17]. Even options for chemical recycling routes for PLA are possible and have been tested [18].

### 2.2. Polyhydroxyalkanoate (PHA)

Polyhydroxyalkanoate (PHA) comprises a family of biopolymers that occur in nature but can also be produced by the fermentation processes of sugar-containing feedstock—in most cases, the glucose obtained from corn starch—in industrial settings using a wide range of microorganisms, such as bacteria, yeast, or fungi. To obtain a plastic material from these processes, the polymer needs to be extracted from the bacterial cells, purified, and finally compounded [6]. Some PHA types are already produced on an industrial scale, for example poly-3-hydroxybutyrate (PHB), poly-hydroxybutyrate-co-hydroxyvalerate (PHBV), or polyhydroxy-butylhexanoate [19]. The properties of PHA depend on the distance between the ester groups in the molecule, the molecular weight, and the functional groups in the side chains [20,21]. PHAs are used in pharmaceutical and medical applications, but are increasingly applied in agriculture, i.e., mulch films, and packaging [22]. In packaging, PHAs are rarely used as pure materials but are blended with other biodegradable plastics, such as PLA or polycaprolactone (PCL), or act as an additive to improve biodegradation properties. PHAs have been the subject of special interest because some of them provide outstanding biodegradability properties and have been shown to biodegrade even in marine environments. [23] However, marine biodegradability is not necessarily a property that is desirable to be communicated to consumers, as it might easily lead to unwanted littering behaviour. Therefore, recyclability and industrial or home compostability should be considered preferable EoL options for packaging made of PHAs.

### 2.3. Biobased Polyethylene (bioPE)

Biobased polyethylene (bioPE) is made by polymerising ethylene that has been obtained through the dehydration of bioethanol. BioPE has been on the market for several years now and is predominately sourced from Brazilian sugarcane and bagasse. However, any source containing sugar- or polysaccharide could potentially be used as feedstock, as route to produce bioethanol from, e.g., corn or sugar beet, and even bioPE from cellulosic feedstocks, are already well known [24,25,26]. Recently, PE made from bio-naphtha, which is produced by the hydrotreatment of plant oil waste, has gained commercial interest. Tall oil, a residue from paper pulp production and also palm oil waste, for example, is currently used as feedstock for bio-naphtha [27,28,29]. The share of biobased content of this kind of bioPE does not necessarily reach 100% and is therefore currently often marketed as “bio-attributed”. A third option to produce bioPE is to produce the PE directly using certain microorganisms, but this technology is still at the research and development level. BioPE has the same chemical and physical properties as conventionally sourced, fossil-based PE and is, similar to its fossil-based counterpart, available as HDPE, LDPE, and LLDPE. Providing a much better carbon footprint, it can be used in the same applications, for example in various packaging, carrier bags, or shrink films, and can, at the end of its material life cycle, be recycled together with conventional PE in the respective recycling streams without any problems [26].

### 2.4. Biobased Polyethylene Terephthalate (bioPET)

Another drop-in bioplastic that has gained a lot of commercial interest in the past years is biobased polyethylene terephthalate (bioPET). The bioPET currently on the market has, in most cases, a biobased content of only up to 30%, as only one building block of the polymer, monoethylene glycol (MEG), can be produced from renewable sources, such as sugarcane [30]. The process route for bio-MEG is quite costly, starting from bioethanol, over ethylene, ethylene oxide, and ethylene carbonate. However, there have been developments to decrease costs by producing MEG directly from sugars [31,32]. The second PET building block, terephthalic acid (TPA), has proven to be more difficult to obtain, as is the case for most renewable aromatics, which is why 100% biobased PET is found to a lesser extent on the market. Nevertheless, there has been ongoing research and development to produce biobased TPA from different sources, such as limonene, bio-paratoluic acid, or bio-paraxylene obtained from glucose using cornstarch as feedstock [33,34,35,36,37]. Consequently, the first bioPET bottles with 100% biobased content have been introduced to the market [38,39].

Similar to its conventional counterpart, biobased PET is used for both technical applications, i.e., fibres and packaging, and mainly for beverage bottles. Like other drop-in bioplastics, bioPET can be recycled easily in the corresponding recycling stream, as its chemical and physical properties are exactly the same as those of fossil-based PET.

### 2.5. Paperboard

Paper is a very traditional renewably sourced material with a long history; there have been many efficiency and sustainability improvements over the years as well as recent innovations especially in terms of barrier coatings that can be applied on paper [40,41]. Some of these are discussed later in this paper and can support the replacement of conventional plastic packaging by fibre-based solutions.

The principal materials used in paper and paperboard packaging include kraft, cardboard, and paperboard, but also specialty papers, such as greaseproof, glassine, cellophane, and moulded pulp. Cardboard has a grammage generally above 200 or 225 g/m^2^, whereas paper has a grammage below 200 g/m^2^. Moreover, most generally speaking, cardboard is multi-ply, whereas paper is single-ply [42].

In general, the pulp obtained is differentiated according to the pulping processes used [43]. In addition, a classification can be made according to the raw material used. If the pulp comes from softwoods, such as pine, larch, or spruce, it is called long-fibre pulp and has average fibre lengths between 2.5 and 4.5 mm. If, on the other hand, the raw material used is hardwood, such as eucalyptus, birch, beech, and poplar, it is referred to as short-fibre pulp, as the cellulose chains are arranged in fibres of average lengths between 0.7 and 1.6 mm [44].

The type of used fibres, the pulping process, type of paper machine, and number of plies distinguish paperboard grades. Different performances can thus be created by combining these material and process variables. Whereas kraft is strong and keeps its dark colour, bleaching or the application of a clay coating is used to obtain a lighter colour and improve the printing properties of the top plies [42].

The main grades are among those using (i) virgin fibres, such as CUK (coated unbleached kraft), SUS (solid unbleached sulphate), SBS (solid bleached sulphate), and FBB (folding boxboard GC); and (ii) secondary fibres, such as WLC (white lined chipboard) or CRB (coated recycled board). SBS and FBB are the most premium grades. They are bleached and have a white surface that may be coated with mineral pigments but differ by the internal combination of pulp types in the successive plies, leading to different yields and weights. SUS is a strong and lightweight kraft board with its main applications in beverage carriers, packaging for frozen foods, detergent boxes, and filter frame structures requiring high strength. WLC as recycled board with shorter fibres may to some extent not meet the mechanical performance standards of the most demanding applications and has been subject to some legal limits in its use for direct food contact depending on the packed product [11,45].

Worldwide, more than 187 million tonnes of pulp were produced in 2018, of which about 62% came from the densely forested areas of North America and Europe. The largest net consumers were China and the European Union (EU) [46]. Fibre-based packaging represents ~38% of total packaging in Europe, followed by plastics (~35%), and has a CAGR of ca 2.5%. Paperboard is the most circular packaging material, as, on the one hand, it is sourced from virgin fibers that derive from sustainably managed forests and are typically obtained as a by-product of the construction industry. On the other hand, it is also made from secondary fibers and currently has a recycling rate of 82% in Europe [47]. There are well-established applications for recycled fibres from consumer packaging and a large fraction is used to produce corrugated board.

### 2.6. Moulded Pulp

Moulded pulp was traditionally produced from recycled paperboard or paper, for example to make egg cartons. Such moulded pulp is recyclable in the paper stream but also compostable and biodegradable. Generally lacking gas barrier properties, it has been for a long time mainly used as protective packaging. However, there have been new developments both in terms of its process and in terms of materials, e.g., applying thin polymer films or barrier coatings that expand the fields of applications of moulded pulp to include ready-meal trays, punnets, clamshells, jars, or even bottles. The high growth of the market for moulded fibre products is also related to the substitution of plastic with fibre-based packaging which has a greater eco-friendly appearance; however, its pricing can sometimes hinder this transition. Recent studies have also investigated how such innovative moulded pulp solutions can reduce the carbon footprint of packaging versus conventional plastic counterparts [48,49].

Moulded fibre products nowadays range from low-value products to premium high-value products. The selection between virgin fibre, which may be needed in terms of safety for new food contact applications, and recycled fibre, as well as the ability to add functionality and/or further treatments to products adds variability. There are several different end-uses for moulded fibre packaging. Protective and industrial packaging is the largest end-use in terms of volume but food service and food packaging are also significant applications, accounting for 40% of the total [50]. Other end-uses include consumer electronics packaging, home appliances, medical applications, and other non-food packaging.

As opposed to other formed packaging, such as pressed trays, moulded fibre or moulded pulp packaging generally starts from a non-flat slurry of fibres that is then formed into its 3D shape. New categories of processes have emerged generating different wall thicknesses and properties.

**Figure 2:**
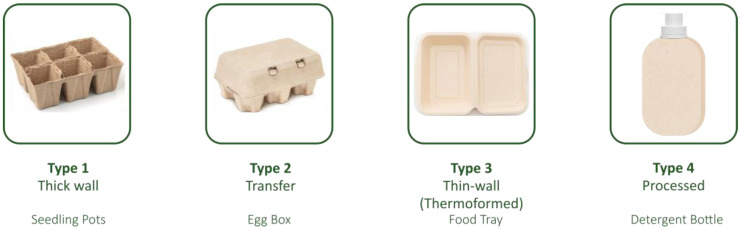
Four different types of fibre moulded products.

Figure 2 shows the main categories of moulded fibre products depending on the wet production technology [50]. Dry production technologies are also emerging and are claimed to have improved productivity and a better environmental profile.

## 3. Biobased Coatings and Adhesives for Multilayer Packaging

Multilayer packaging structures are widely used in the packaging industry, as these structures are able to achieve low oxygen permeability. The protection against oxygen is essential not only for sensitive and fresh food but also for cosmetic and pharmaceutical items, in order to ensure a sufficient shelf life. Insufficient oxygen protection can lead to various issues, such as colour or taste deviation, the oxidation of fats, the undesired growth of microorganisms, or the degradation of nutrients. To address this, the most widely used oxygen barrier materials are ethylene vinyl alcohol co-polymer (EVOH) or polyvinylidene dichloride, which are derived from petrochemicals [51,52]. However, these polymers have the downside of neither being biodegradable nor being biobased. Moreover, conventional multilayer structures are difficult to delaminate and, therefore, difficult to recycle. Consequently, research on sustainable packaging materials, which show appropriate barrier properties while still being able to be removed, is in progress. A selection of these advances is summarised in the following chapters.

### 3.1. Whey-Protein-Based Films and Coatings

Due to current efforts to develop more sustainable packaging materials for the production of packaging materials, proteins from the residual products or by-products of the food industry are becoming increasingly important. The growing interest of the public and thus also of politicians in packaging materials based on renewable raw materials and biodegradable packaging materials is also leading to increased research activities on proteins such as wheat gluten, soy protein, casein, and whey protein for their use as possible resources for packaging materials.

Protein-based films and coatings can serve several functions. They can be applied by different techniques to improve the packaging-relevant properties of packaging materials, such as barrier properties, or they can be used as an adhesive to allow the lamination of different materials [53].

A very extensively studied protein in terms of its potential in packaging applications is whey protein, a by-product of cheese production. Whey-protein-based formulations have already been shown to be suitable for the production of edible coatings and films. Although these films exhibit only a medium water vapour barrier due to their hydrophilic character, they have excellent oxygen barrier properties [54]. Another advantage of whey protein is its availability. As a by-product of cheese production, whey protein is produced in large quantities [55].

Purified whey protein concentrates (WPC) or even highly purified whey protein isolates (WPI) can be used to form whey protein-based films. In order to form coherent and non-brittle coatings, different plasticizers are incorporated into an aqueous film-forming solution. The final properties of the resulting films and coatings vary with the proteins and additives used. In particular, the concentration of plasticizers, chemical agents, or the addition of lipids or salts affects the resulting properties [40].

Whey-protein-coated film can be laminated to other packaging films to create recyclable multilayer structures as developed in the European funded projects Wheylayer, Wheylayer2, and Thermowhey. These multilayer structures can be used in additional conversion processes, such as thermoforming or form-fill-seal processes, in order to create different packaging items with tailored properties. The application of whey-protein-based coating formulations has been proven to work in pilot scale in roll-to-roll coating and lamination lines. The films produced were found to possess exceptional mechanical properties, as well as effective barrier properties against gases and ultraviolet (UV) light [56]. Whey-protein-based coatings in multilayer structures have the potential to replace synthetic oxygen-barrier layersincluding EVOH. This bio-based and biodegradable coating provides an alternative to organic fossil-based barrier materials [56]. A recent study has shown that whey protein-based multilayer packaging films can provide similar functionalities as those of multilayer packaging films with inorganic barrier coatings [57]. The combination of protein-based films and coating with inorganic coatings could be an interesting approach to further improving the functionality, such as the barrier properties, of novel sustainable multilayer packaging films. 

The application of whey protein coating on paperboard has also been the research subject of several studies. These studies demonstrated that whey proteins decrease water vapour and oxygen permeability and increase the oil resistance of paper(board), whereas the grease resistance of the coatings depends on the plasticiser used [58,59,60,61]. A more recent study aimed to further improve the barrier properties of whey-protein-coated paperboard by applying a new WPC–beeswax–sucrose coating [62]. This combination resulted in decreased water absorption and decreased water vapour permeability compared to WPC-coated paper without beeswax. Combining protein coating with lipids or lipophilic compounds seems to be an interesting strategy to achieve further improvements. A different technology allowing the combination of proteins and lipophilic components is described in Section 3.1.1.

#### 3.1.1. Chemical Modifications of Proteins

The chemical modification of proteins for edible films and coatings can involve reactions with different chemical agents [63]. Alkylation, acylation, acetylation, and succinylation are some selected chemical reactions that can take place in protein-based film-forming solutions. However, in case of food packaging materials with direct food contact, the recent legal situation regarding food contact compliance needs to be considered very carefully; therefore, several modification pathways are not suitable, as the potential chemicals are lacking food contact compliance. Chemical grafting, meaning the application of fatty acid chlorides by an acylation reaction, could potentially gain importance for whey protein modification. Here, alkyl chains covalently bond to protein chains and can act as internal plasticizers. Comparable to the effect of external plasticizers, the intermolecular interactions between the protein sides chains are reduced, resulting in changed properties of the protein films and coatings [63,64].

A highly material-efficient method to increase the water vapour barrier and repellence properties of biobased films and coatings is chemical grafting technology using fatty acid chlorides, e.g., palmitoyl chloride, to monomolecularly graft a hydrophobic nanoscale layer on hydroxyl-group-containing surfaces [65]. Experiments at Albstadt-Sigmaringen University first showed that the water vapour barrier properties of protein-based films could be altered by this grafting process (data not published). Fatty acid chlorides can be applied by a transfer method that was developed for the surface grafting of polyvinyl-alcohol-based films [66,67,68]. The fatty acid chlorides react with the hydroxyl groups of the substrate surface. The main advantage of this grafting process is its material efficiency. In addition, the fatty acid nanoscale grafting provides hydrophobic surface properties [66], leading to an improved easy emptying behaviour of several packed goods, such as high-viscosity food products. These repellence properties are comparable to those of emerging non-biobased and non-biodegradable solutions on the market.

### 3.2. Polyhydroxyalkanoate (PHA)-Based Coatings

PHAs constitute the carbon and energy storage material of certain bacterial species produced under carbon excess and nutrient limitation conditions [69,70]. They are aliphatic polyesters that can be classified according to their monomer size as short-length PHAs, consisting of three to five carbon monomers, and medium-chain-length (mcl) PHAs with six to fourteen carbon monomers in the 3-hydroxyalkanoate units [71].

The ability to manipulate and modify the cellular system through metabolic and genetic engineering tools has paved the way for the creation of PHAs that are rationally designed [72,73]. This has been demonstrated by controlling the carbon flow through the beta-oxidation pathway, which results in obtaining a higher percentage of shorter monomers in the PHA when the repressor PsrA (PsrA: *Pseudomonas* sigma regulator) is deleted [74] or longer monomers when the beta-oxidation pathway is slowed down [75].

Depending on the monomer composition and the length of the side chain, different PHA properties are obtained in regards to hydrophobicity, melting point, glass transition temperature, and degree of crystallinity, as well as a wide variety of mechanical properties from rigid and highly crystalline to flexible, amorphous, or elastic [20,21]. Structural diversity is critical to defining potential applications, as the biological, thermal, and mechanical properties of the resulting polymer depend on the monomer composition and molecular structure of the polymer. Long-chain PHAs are generally water-insoluble and resistant to hydrolytic degradation due to their high hydrophobicity [76]. They possess excellent film-forming properties, a higher water vapour barrier than other biopolymers, good UV resistance, and poor resistance to acids and bases [77]. Yeo et al. (2018) [78] summarized that adjusting the ratio between hydroxyvalerate (HV) and hydroxybutyrate (HB), which can be achieved by the manipulation of the growth media, can alter the properties of the film A high proportion of polyhydroxybutyrate (PHB) lead to a strong and rigid material, while polyhydroxyvalerate (PHV) increases flexibility and toughness.

Medium-chain-length (mcl) PHAs are considered promising candidates for packaging applications due to their high elasticity, hydrophobicity, low oxygen permeability, water resistance, and biodegradability [79]. Using mcl-PHA in blends with poly(3-hydroxybutyrate-co-3-hydroxyvalerate) PHBV or PHB results in an improved elongation at break and decreased tensile strength compared to those of neat PHBV or PHB films [80,81]. In the case of PHB, the O_2_ permeability is decreased by 38% upon blending with mcl-PHA. Comparing the aforementioned PHB/mcl-PHA blended films with neat mcl-PHA films, the reduction in O_2_ permeation is even more prominent [81]. PHA coated on cellulose nanopaper significantly improved the surface hydrophobicity [82]. In another study published by Pérez-Arauz et al. (2019) [83], cast films of blended PHA, which were rich in PHB with low amounts of PHBV and mcl-PHA, showed an increase in the elongation at break in comparison with that of homopolymer PHB, and a decrease in melting temperature. Moreover, regarding water vapour permeability, the blended cast films had the same order of magnitude as the value reported for PET. These properties further indicate their application potential in the food packaging sector [83]. Nevertheless, the properties of the polymers need to be diversified to be applied in different fields [84].

PHAs have been processed using different techniques, such as extrusion and injection [85,86], compression moulding [77], thermoforming [86], solvent and spin casting [87], and extrusion coating [88,89,90]. The exploitation of PHA as a coating is still hampered by difficulties in processing mainly due to its crystallization behaviour and its narrow thermal processing window [91]. In general, these issues can be solved by adding additives or blending with other polymers. Blending PHB with natural raw materials or other biodegradable polymers, including starch, cellulose derivatives, lignin, or PLA, can improve ihts poor mechanical and thermal properties, thereby extending its as a coating material for food packaging applications on paper or cardboard [92,93,94,95].

Noda et al. [96] reported that 10% of PHA to PLA improved the toughness and elongation at break. Moreover, the tensile strength of a PHB–starch blend at a ratio of 30:70% significantly increased compared with that of the virgin PHB, leading to a substantial reduction in costs [71]. Nevertheless, blending can be complicated by the interfacial interactions between the polymers, so modification by grafting or copolymerization is needed. Willett et al. [97] used a grafted starch/glycidyl methacrylate copolymer leading to improved mechanical properties of PHBV.

To further enhance its barrier and mechanical properties and overcome the difficulties regarding its processing properties, PHA could be combined with additives and fillers, as their addition affects the crystallization behaviour. The critical point in this case is to achieve a homogeneous dispersion of these fillers. Furthermore, these fillers can act as nucleating agents to promote crystallization in order to improve barrier properties and reduce the brittleness of the coatings. Nanofillers with different morphologies and chemical natures, such as natural clays, micro- and nanofibrillated cellulose, and cellulose nanocrystals, can be considered [98]. El-Hadi [99] has shown that the integration of clays in PHB improves the barrier properties against water vapour and oxygen. The integration of nanocellulose led to a significant improvement of 70% in the water vapour barrier properties with 15% of CNC in PHBV according to Yu et al. (2014) [100]. Simultaneously, the mechanical and thermal properties were also enhanced [100]. Coatings consisting of plasticized PHB and cellulose nanocrystals were found to enhance the performance of paperboard not only in terms of the water vapour barrier but also regarding its tensile properties [101]. In conclusion, blending and incorporating additives provide the opportunity to obtain favourable barrier properties and improve mechanical properties.

Nevertheless, the dispersion and solvent casting processes continue to be a challenge for PHB. This process involves dissolving the polymer in a suitable solvent to obtain ultra-thin films with a high optical clarity. Chloroform is one of the most compatible solvents for PHB [102,103]. Thus, PHB has been coated on paper by solvent casting using chloroform as a solvent, resulting in a reduction in moisture and water absorption along with improved tensile properties [104]. However, as chloroform is one of the chemicals that is the most damaging to the environment and human health [105], there is a need to find risk-free solvents. Anbukarasu et al. [106] demonstrated the possibility of using acetic acid as an alternative to chloroform, obtaining films with comparable properties. PHB coatings can also be obtained by aqueous polymer dispersion processes, but PHA continues to pose a challenge due to unsatisfactory performances and relatively high costs [107,108]. Future developments are necessary in this area mainly for paper coating in order to pave the way for a sustainable and green future.

Besides solvent-based coatings, PHAs can also be applied as waterborne coatings using the patented plastisol method [109]. This plastisol consists of PHA powder, plasticizer, and water, which are mixed into a homogeneous, coatable paste. This paste can then be coated via knife coating, rod coating, or screen-printing on a range of substrates, such as textiles, paper, or foils [109]. After the application of the plastisol, the coating is heated above the melting point of the used PHA to evaporate the water and fuse the PHA and plasticizer into a homogeneous coating. Visually, the coatings are colourless and transparent. The coatings show excellent abrasion resistance, good flexibility, and promising barrier properties [109].

Using a combination of β-oxidation pathway-modified strains and growth on selected carbon sources, PHA can be produced with a tailored monomer content, yielding PHA with desired properties for specific applications. PHAs with more or less similar structures, but different physical properties due to their lower glass transition temperature compared with PLAs, are referred to as second-generation bio-polyesters. Overall, we highlight here that PHAs might be a better candidate for thin film manufacturing not only due to their synthesis by microorganisms and the biodegradability of unmodified PHAs in several environments, but also because of the significant variability in their microstructure that in turn provides a wide range of properties. PHA has found many potential applications in a wide variety of fields thanks to its biocompatibility, biodegradability, and low permeability against H_2_O, CO_2_, and O_2_. The present barrier to its bulk use is its cost. This is partially connected with the manufacturing route and partly the production scale. Consequently, a key factor in the spreading of biobased polymers in food packaging is the development of analogous continuous processes for manufacturing at a reduced cost and to a pre-defined quality. Another key factor is the production of polymer resins that can readily be processed into film using existing industrial machinery, only necessitating a minor modification of the production plant. The potential of this polymer type in the packaging sector is excellent.

### 3.3. Biobased Adhesives

Multilayer materials for packaging applications are structured by bonding two or more substrates through an adhesive. Substrates in flexible multilayer packaging are typically fossil-based polymers, such as PE; PP; PET; polyamides (PA); biobased polymers, including PLA, PHA, bioPE, and bioPET; fibre-based materials, i.e., cardboard, paper, barrier coatings, such as EVOH; or inorganic barrier materials, e.g., aluminium [110]. The multilayer materials can be distinguished between laminates, i.e., adhesives applied on the full area of the packaging, and seams, i.e., adhesives applied over a partial area of the packaging for sealing. The area of adhesive application has a big impact on the adhesive forces that can be developed [111].

Adhesives in general are classified according to their intended function, their chemical composition, method of curing, physical form, and their applications [112,113]. In terms of sustainability, a distinction between synthetic or natural adhesives has become more and more common.

#### 3.3.1. Adhesive Types and Potential of Biobased Adhesives

Synthetic adhesives include a broad spectrum of adhesives and can be sub-classified according to their method of curing, which comprises mainly the following three mechanisms: (1) a loss of solvent or loss of water, i.e., solutions or dispersions; (2) cooling from a melt, i.e., hotmelt adhesives (HMAs); and (3) curing by a chemical reaction, i.e., polyaddition or radical polymerisation.

For the first type, the solvent subsequently evaporates to give the final joint. In terms of environmental safety, using water instead of organic solvents has become more popular recently. Solvent-based adhesives can be based on polyvinyl acetates, polyvinyl alcohol, ethylene vinyl acetate copolymers (EVA), polyurethanes (PU), acrylates, and natural and synthetic rubber [110,114,115,116]. HMAs, on the other hand, are polymers or polymer mixtures that join two substrates by cooling from a melt. The joint formation is achieved very fast. Therefore, HMAs are usually chosen for processes that require a high throughput. HMAs are thermoplastic polymers, such as PA, saturated polyesters, and EVA [117]. The third type of adhesives are those cured by a chemical reaction. They can be separated into one-component reactive adhesives, in which the crosslinking reaction is triggered by an external impulse; and two-component reactive adhesives, in which the two reactants are mixed shortly prior to their application [118]. One-component adhesives can be PU, silane adhesives, and cyanoacrylates, which are cured by water; condensation resins, such as phenol-, urea- or melamine formaldehyde adhesives, which are cured by high temperatures; or acrylates, which are cured through UV light. Two-component reactive adhesives can be epoxides, PU (addition reaction), or methacrylates (a radical polymerisation reaction started by an initiator compound) [119]. A separate class of adhesives are pressure-sensitive adhesives (PSAs). They do not harden, but retain their tackiness, which is caused by non-covalent interactions with the substrate, throughout their service life. Therefore, they are used in adhesive tapes and labels. PSAs are mainly based on acrylates, rubbers, and UV-curable polymers [120].

The adhesive has to address many technical needs, such as the bonding strength between substrates, including printed ones, heat resistance, and chemical resistance [121]. It is a key factor in maintaining the packaging clear and shiny, preserving the marketing design given by the printed film. Above everything, the adhesive must be safe for consumers and respect all food legislation applied at a country or regional level [121]. Reactive PU adhesives that form a cured PU network as the adhesive layer may fulfil these requirements. These adhesive systems are applied by a roller process with or without a drying step depending on the solvent contents [122]. A PU adhesive is generally formed by the polyaddition reaction of a hydroxylated component with an isocyanate component. The hydroxylated component can vary widely, ranging from straight polyesters and PU pre-polymers to polyether polyols. The same goes for the isocyanate component, which ranges from PU pre-polymers based on either polyester or polyether to aliphatic or aromatic isocyanates [122]. These systems with many variables give a high degree of freedom for the formulation, customisation, and optimisation of performance and efficiency. The properties of PU could be tuned by the appropriate selection of these starting materials. Some biobased polyols, including polyester polyols [123,124], estolide polyols [125,126,127,128], polylactic polyols [129,130], and natural oil polyols [129,130,131,132,133,134], have been attracting interest lately; thus, biobased dicarboxylic acids and diols/triols have already been developed and are commercially available as potential substitutes for their petroleum-based counterparts. However, the choice is limited by regulations and current industrial potential.

Except for some aliphatic isocyanates, such as pentamethylene diisocyanate [135,136] and derivatives, the number of biobased isocyanates is extremely limited. The PU adhesive could be supplied either as a solvent-based product or as a solvent-free one. Solvent-based PU adhesives can cover all the needs of the market from general purpose applications, such as packaging for snacks, to highly demanding applications in the retort and packaging of chemically aggressive products. Solvent-free adhesive systems, 100% solids in this case, are generally not efficient enough for highly demanding end-use when compared to solvent-based ones [137,138].

Natural adhesives are manufactured from naturally occurring materials, such as animal or agricultural products, including starch, casein, animal glue, fish glue, blood glues, and natural rubber. However, besides originating from renewable resources, they have further advantages compared to fossil-based synthetic adhesives [133]. By using natural building blocks with different functionalities, e.g., unsaturated or epoxidized oils, peroxidized starch, itaconic and cinnamic acid derivatives, and terpenes, the molecular architecture of biobased adhesives can be different to that of petroleum-based adhesives, resulting in new interesting properties, as summarized by Heinrich (2019) [133]. For example, the introduction of lignins with high chemical functionality can increase the curing speed and strengthen the adhesive bond. Furthermore, water resistance can be promoted by the introduction of long alkyl chains from vegetable oils. New functionalities such as those deriving from nanocellulose, which can act both as a binder and as a structural reinforcement, can be introduced into biobased adhesives [133].

The use of renewable feedstock for the development of novel adhesives is an incentive for the circular economy. Furthermore, next to natural building blocks, waste feedstock can be an alternative to reduce the carbon footprint and to promote the circular use of plastics [133]. Thus, oligomers and monomers obtained from the degradation of biobased polymers, such as PLA, can be used to develop novel adhesives. Commercial PLA cannot be used as an adhesive because of its high molecular weight and its high transition glass temperature. However, the presence of hydrolysable ester bonds and the ability to be degraded by enzymes make its degradation products interesting for the development of biobased adhesives. Recently, lactic acid oligomers (OLAs) have been used in different adhesive formulations. Wendels et al. (2022) [139] prepared biobased polyurethane sealants for tissue adhesive applications, based on an isocyanate-terminated pre-polymer and a chain extender. In order to bring controlled variations on the final macromolecular architectures, they prepared different prepolymers based on poly(3-hydroxybutyrate) diol oligomers and poly(lactic acid) diol oligomers, obtaining different physico-chemical and mechanical properties. Moreover, the final adhesives exhibited good biocompatibility, with limited cytotoxicity [139]. OLAs were also used in the synthesis of renewable pressure-sensitive adhesives, by their copolymerisation with epoxidized soybean oil [140]. The carboxyl and hydroxyl end-groups of OLAs are in fact able to open the epoxy rings of the oil and to form polymer networks with good adhesion properties.

#### 3.3.2. Protein-Based Adhesives

Adhesives derived from proteins offer several advantages, such as a wide applicability in terms of their processes and substrates, non-toxicity, safety for indirect food contact, biodegradability, easy clean up with water, and cost effectiveness [141]. The drawbacks of protein-based adhesives are their poor water resistance, the low adhesion with non-polar-based substrates, such as polyolefins, and their limitations regarding direct food contact or exposure to low temperatures, i.e., freezers.

These macroscopic properties can be related to the chemical structure of proteins. They have a primary, secondary, tertiary, and quaternary structure, which depends on the outer conditions and can be modified to change the protein’s properties. As a natural biopolymer, proteins have a high degree of free functional groups resulting from the natural amino acid profile, such as the carboxy, amino, thiol or hydroxyl groups. These functional groups, unlike other biopolymers, are relatively easy to chemically modify, and, therefore, the properties of the protein can be adjusted for the target application.

Casein, obtained from cow’s milk, precipitated by acid and solubilized again by alkali, is the main protein for adhesives. Adhesives based on casein were traditionally used for laminating timber, since the wooden pieces could easily be reassembled and repaired by releasing the brittle joint. Casein has been also used ever since to attach labels to glass bottles due to its good adhesion on glass in cold or wet environments and the ease of removing it under hot water prior to recycling or refilling [121,142]. Further crosslinking can be achieved by metal salts containing bi- or trivalent metals, for example calcium or aluminium. To influence the setting time, casein glues contain urea. All formulations need a preservative and a defoamer [122].

To improve its resistance against water, various strategies of protein modification have been reported. Chemical crosslinking involves the modification of the tertiary and quaternary structure, for example, creating denser networks, which are less prone to water [143]. Another strategy, in which soy protein is self-crosslinked with pre-hydrolysed carbohydrates from soy flour, has been shown to increase moisture resistance and improve shear strength by almost 100% [144,145].

The interfacial adhesion of soy protein can be improved by inorganic fillers, such as calcium carbonate, which interact with the functional groups of the protein [146]. The formed nanocomposite has been used to bond plywood samples. The shear strength increased from 1.7 MPa to above 5 MPa [146].

The hydroxyl- and amine functional groups of soy proteins have been covalently bonded to traditional crosslinking agents, such as poly-methylenediphenylisocyanates for obtaining a similar shear strength to that of commercial adhesives [147], a bisphenol-A-based epoxy resin to increase the shear strength by 55% when hydrolysed [148], or urea-formaldehyde resin to increase the shear strength by 150% [149]. Blending proteins with commercial adhesives can also improve their performance, as demonstrated in a formulation of urea formaldehyde resin with soy protein [150]. Using 25% soy protein increased the internal bond strength of glued cardboards by a factor of two to three.

Non-polymeric crosslinkers for proteins have also been reported. These non-polymeric crosslinkers include epoxy-silane coupling agents that have double the shear strength of unmodified soy-protein adhesives [151]; glutaraldehyde, which increases the molecular weight of bacteria protein by a factor of six [152]; and L-3,4-Dihydroxyphenylalanine, which increases the shear strength of soy protein by a factor of three [153]. Crosslinking soybean protein with soybean-derived daidzein results in a nearly 100% biobased adhesive with an improved shear strength and increased mildew resistance [154].

The modification of the tertiary structure of soy protein by disrupting intramolecular crosslinks through guanidine hydrochloride, sodium dodecyl sulphate, sodium hydroxide, or urea increased the shear strength of soy protein slightly, but showed the reverse effect for cottonseed protein [155]. Likewise, enzymatic hydrolysis, followed by heating to 50–90 °C, improved the bond strength and water resistance of a wheat gluten adhesive [156]. Changing the pH value can either increase or decrease the adhesive strength [157]. In general, the highest shear strength of protein adhesives is near their isoelectric point. The solubility in water also is lowered by increasing the hydrophobicity caused by the stronger interaction between protein chains [158,159]. The susceptibility of proteins to hydrolysis and their degradation upon exposure to a high humidity during service needs to be addressed, but can at the same time advance the recyclability of the blended products such as adhesive tapes, which can potentially be made fully biodegradable [160].

As an example for a HMA, a mixture of coconut oil, PCL, and soy protein isolate was prepared and its mechanical and thermal properties were analysed [161]. The addition of 40% soy protein increased the softening point from 60 °C to above 75 °C, while the tensile strength decreased from 11 MPa to 2.9 MPa.

### 3.4. Further Biobased Multilayer Approaches

Besides the intensively studied whey protein and PHA-based coatings described in Section 3.1 and Section 3.2, respectively, several other biobased coatings are under investigation for applications in multilayer structures. Besides animal-derived materials, such as fish gelatine, casein, chitosan, and others, plant-based proteins and coatings, including soy protein and zein, also have shown great potential for application in laminates [40,162,163]. Besides terrestrial plants, macrophytes (aquatic plants) can be considered a potential protein source for packaging applications [164].

Another approach for gas-barrier coatings has been patented for combining natural proteins and natural polysaccharides with synthetic polymers as structuring agents and metal oxides as reinforcement agents. The applicable proteins comprise gelatine, wheat gluten, casein, zein, or whey proteins, whereas pectin, cellulose, chitosan, alginates, carrageenan, guar gum, or xanthan gum can be used as polysaccharidic materials [165].

Side stream products, for instance from food processing, are of high value as raw materials. For instance, a moulded and fluff pulp material was recently patented to be useful as a biodegradable packaging material. The applicants for the patent filed a method to use the aliphatic polyesters present in the moulded or fluff pulp and a cellulose-based laminate layer to produce a compostable food packaging unit [166,167]. Another recently published patent describes a method for obtaining concentrated protein-rich phases from residues of bioethanol production [168]. The obtained by-products can then be used for the production of self-compostable films, coatings, and rigid plastic after further modification and purification [169].

Outside Europe, several patents have recently been filed in the field of food packaging. These innovations include biodegradable packaging solutions, such as composites consisting of more than five components [170,171,172,173]; multilayers combining nanocellulose with guar gum or chitosan [174,175]; and mixed—biobased and fossil-based—biodegradable multilayer structures consisting of polybutylene adipate terephthalate (PBAT), polypropylene carbonate (PPC), PLA, and PHA [176]. Moreover, a recently published patent describes biobased barrier coatings using prolamins, such as zein, hordein, gliadin, and kafirin, together with oilseed-derived polyol fatty acid esters [177]. A broader approach combines biobased plastic—mainly drop-in solutions—with recycled materials and conventional, fossil-based plastics to achieve polymer compositions with a lower CO_2_ footprint [178].

Besides the aforementioned multilayer approaches, another approach, which is not included in this review article, is the use of biocomposites. A variety of biocomposites have been and are still being extensively studied regarding their potential application in (food) packaging [179,180,181,182].

## 4. Recycling, Biodegradation, and Upcycling

The destiny of post-consumer bio-plastics is a subject of paramount importance in designing new industrially viable and sustainable products. The chemical complexity of new biobased and biodegradable materials on the market substantially widens the possibilities for their EoL, requiring new definitions and new strategic planning [183]. A recyclable material can be collected and subsequently physically or chemically treated to obtain a second-generation material presenting the same chemical composition and, in the best-case scenario, restored performances. This method can be performed under the premise that the polymer does not undergo degradation during the recycling process. Biodegradation was previously introduced and refers to the microbial degradation of the material with the production of water, CO_2_, and biomass. Such an approach sees the complete destruction of the macromolecule and should be preferred when an application prevents a suitable recycling method. The upcycling approach is a relatively new concept and regards the combination of controlled chemical/enzymatic degradation along with a modification step to create a second-generation material presenting brand-new performances, aiming at higher-value applications. However, such a variety of scenarios can be concretely performed only after the proper recovery and sorting of postconsumer plastic.

### 4.1. Identification and Sorting

Within the circular economy paradigm, there is increasing interest in pushing the EoL of plastics towards more recycling to avoid resource depletion and leakages in terrestrial, freshwater, and marine environments. Only 31% of plastic packaging is currently recycled, mainly limited to rigid packaging, such as PET bottles or other pure PP or PE items. However, an excessive amount, about 30–40%, is still landfilled due to the lack of adequate technologies or to economical unviability [184]. Although the share of bioplastics in the global plastics market remains low at around 1–2% of overall plastics production, as mentioned in the introduction, the global production of bioplastics is reported to have steadily grown and is even forecasted to triple in the upcoming years [1]. With the increasing market share of bioplastics, processing materials at EoL need to be considered [185].

Most post-consumer bioplastics will end in the waste flow together with conventional plastics. Although some biobased plastics are biodegradable and could be biologically decomposed, degradation is not the optimal treatment method for biodegradable plastic waste. Instead, recycling achieves the highest environmental benefits according to life cycle analyses [186]. In addition, with the exception of drop-in solutions, biobased plastics differ from fossil-based plastics and could interfere with the current recycling of conventional plastics, thus hindering the closure of plastic cycles. This is undesirable in view of the current focus on the transition to a circular economy. [187] Hence, a successful identification and sorting of the new biobased materials becomes fundamental for the whole plastic waste recycling process. Despite the fact that biobased plastics as group were not found to pose an overall contamination risk for current recycling practices, each of the biobased plastics must be considered as a potential separate source of contamination. In the case of PLA, its presence, even at low levels, can provably cause a deterioration in the quality of recycled PET. Therefore, in order to said deterioration, contamination of the feed for mechanical recycling should be kept well below 0.1%. [185,187] Hence, it seems necessary to evaluate the impact of biobased plastics on the current recycling of plastics, ensuring that the compatibility with the already established recycling processes for conventional plastics is evaluated and striving for during the development of new biobased packaging solutions.

Most biobased and biodegradable plastics can be processed with conventional waste management options and thus have the potential to be recycled into new products [186,188]. Existing sorting and recycling industrial technologies for conventional plastics are mainly focussed on the separation and recovery of single materials, such as PP or PE, from plastic items containing a unique type of material [189]. On the contrary, the sorting and recycling of multilayers is still under research and development with limited industrial applications [190]. This will also be the case for biobased multilayer materials.

Current waste management technologies for the characterization and analysis of EoL plastics are mainly based on optical spectroscopies. These techniques use different physical phenomena but can be grouped into molecular and atomic spectroscopies according to the information that can be obtained from them. The group of molecular spectroscopies includes Fourier transform infrared (FTIR) spectroscopy, mid-infrared hyperspectral imaging, Raman spectroscopy, near-infrared (NIR) spectroscopy and NIR-hyperspectral imaging, and Terahertz imaging. Laser-induced breakdown spectroscopy and x-ray fluorescence spectroscopy belong to atomic spectroscopies [191].

When it comes to identifying and sorting biobased plastics, it is worth mentioning that some of them—particularly biodegradable ones—can suffer from degradation processes. Theses degradation processes can affect their fingerprints. The influence of bioplastics on the sorting processes of conventional plastics by means of near-infrared (NIR) spectroscopy has also been studied. Using hyperspectral imaging (HSI) technology, Chen et al. (2021) [192] reported that the presence of non-degraded and degraded PLA in lightweight packaging waste in Germany does not influence the sorting process of the main sorted conventional plastics: PP, HDPE, PET, and PS.

#### 4.1.1. Near-Infrared Spectroscopy (NIRS)

Over the past 30 years, on/in-line near-infrared spectroscopy (NIRS) has proven to be one of the most efficient and advanced tools for the continuous monitoring and control of process and product quality in a wide variety of industries. Among the diversity of applications for NIRS, the recycling of plastics has received a lot of attention from industrial communities and also from the governments of different countries [193].

NIRS is a vibrational spectroscopic technique that consists of the interaction between electromagnetic radiation and a material within the wavelength range of 780–2500 nm. The absorption bands seen in this spectral range result from harmonics and combination bands of O-H, N-H, C-H, and S-H stretching and bending vibrations. These bands allow the qualitative and quantitative assessment of chemical and physical characteristics. Therefore, NIRS can be applied to all organic compounds that are rich in a) O-H bonds, e.g., moisture, carbohydrates, and fats, b) C-H bonds, e.g., petroleum derivatives, and c) N–H bonds, e.g., proteins [194].

A basic NIRS setup includes a light source, which is commonly a quartz–tungsten–halogen lamp, beam splitter system, i.e., wavelength selector, sample detector, and optical detector. Additionally, extracting relevant information from spectral data requires requires the application of mathematical and statistical procedures. This processing is known as chemometrics. [195] The possibility to use intact samples, which are directly presented to the instrument without any pre-treatment, is one of the main advantages of NIRS. Depending on the type of sample to be analysed, NIRS spectrometers can work in transmittance and/or reflectance modes [196].

In general, plastic waste products can be separated into two main fractions. Domestic wastes contain only five relevant polymers in large quantities (PE, PP, PS, PET, and polyvinyl chloride). Technical plastics use a broad variety of polymers containing fillers and additives. The plastics from domestic wastes can be reliably identified by the first overtone of the C-H bands between 1600 and 1800 nm. Extending this wavelength range up to 1000 nm, technical non-black plastics can also be identified. In the case of black plastics, the mid-infrared (MIR) spectral range has to be applied as well, due to the reduced penetration depth of radiation into these materials. Additionally, larger amounts of additives, such as plasticizers and flame retardants, can be analysed [197].

NIRS is a successfully technique to identify and thus to classify a number of commonly used plastics, such as PE, PP, PET, and PS, and also biobased polymers, such as PHA and PLA [197,198]. In addition, NIRS has been shown to be a suitable analytical tool for the monitoring of waste recycling [199,200,201].

The identification of plastics from waste in recycling plants requires fast scanning techniques in the millisecond range, especially if many samples are taken for identification of movement. This movement excludes the FTIR system [197], which is an effective tool in identifying plastics off-line, but not on-line.

#### 4.1.2. Hyperspectral Imaging (HSI)

Although punctual measurements with NIR can be useful for the off-line identification of small amounts of plastic waste, in the plastic recycling industry there is usually a large amount of plastic waste to be identified and sorted, thus a NIR-based technology combining spectral fingerprints with spatial information seems more appropriate for recycling processes.

HSI in the NIR region has been widely used for in-line monitoring applications, due to its capability to provide information regarding composition and its spatial distribution [202]. These characteristics have reinforced its use in the solid waste recycling industry.

A basic HSI system consists of a sensitive NIR sensor, which usually is a CCD camera, a broadband illumination source, which often is a tungsten lamp, a spectrometer, which separates the backscattered/transmitted light into its different wavelengths, a computer, and a conveyor belt for sampling when required. In addition, the system requires software support for image acquisition and control, the multivariate analysis of the spectra, and final image processing [194].

**Figure 3:**
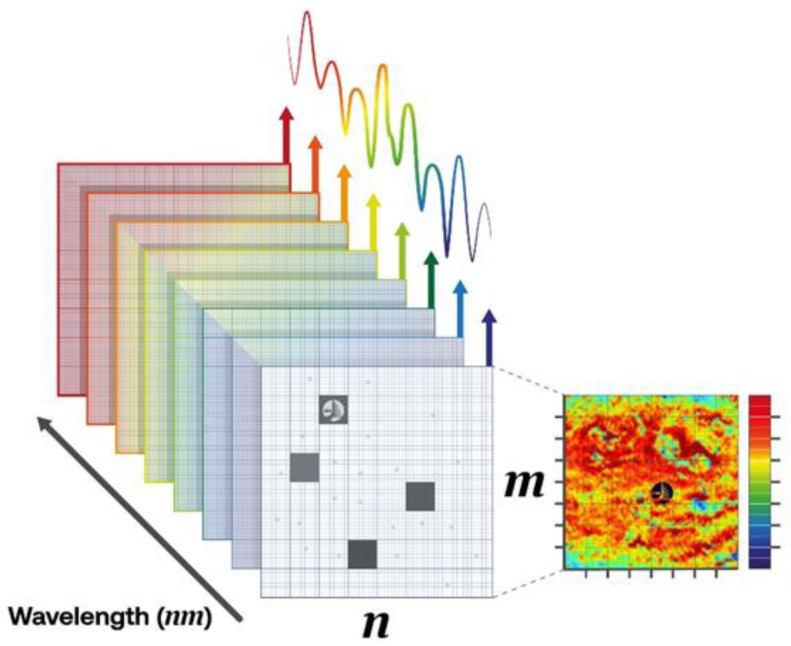
Graphic representation of a hyperspectral cube acquired with the industrial hyperspectral system VISUM^®^ HSI^TM^. Each hyperplane (slide) of the cube corresponds to a spectral channel or wavelength band.

The output of a HSI system is a multispectral image of the sample tested, containing the spatial localization of its interesting features and chemical composition. This data set is generally organised into a hyperspectral data cube (Figure 3). A hypercube is a set of data ordered in three dimensions, two spatial (a plane MN) and one spectral (λ, wavelength). Such a set of data has to be processed using advanced mathematical tools, also known as chemometric techniques, including a multivariate analysis and statistical methods, to extract the most relevant and significant information from the spectral dataset. In this regard, chemometric methods allow the analysis of a large amount of data, identifying the most significant spectral features for discrimination, classification, or prediction purposes.

The use of HSI technology in the plastic recycling industry has been described in a number of publications [202,203,204,205,206,207,208]. In addition, a few studies have been performed regarding the use of hyperspectral imaging for the identification of bioplastics. Ulrici et al. (2013) [204] demonstrated the effectiveness of HSI in the NIR range (1000–1700 nm) coupled with chemometrics to discriminate PET from PLA. Hollstein et al. [198] concluded that novel bio-plastics made of PLA, potato starch, or corn starch were distinguishable from the established, conventional ones by means of HIS-NIR-SWIR (1200–2200 nm). Moroni and Mei [209] developed an effective methodology based on HSI on the NIR region (900–1700 nm) for the separation of two different conventional plastic polymers (PET and PS) and PLA, which is a biobased and biodegradable plastic material, at different phases of their life cycle—in particular, as primary raw materials and urban waste—and with different morphological and dimensional characteristics.

### 4.2. Recycling

Framed in the pivotal fields of sustainable development and the circular economy, the recycling of plastic materials represents one of the most relevant aspects for the development of new products. The possibility of re-using materials for multiple life cycles and a pool of different applications allow us to save a substantial amount of primary material resources as well as energy, while positively reducing our carbon footprint [210]. Today, the main challenge resides in designing EoL options in advance for multiple applications [211].

Several technologies have been developed for the recycling of polymers and plastics, including mechanical processes, i.e., shredding, grinding, and melting [212]; chemical processes, i.e., hydrolysis, alcoholysis, and thermal depolymerisation with catalysts [213,214]; and the depolymerisation of (bio)polymers using enzymes [215].

The recycling of biobased polymers allows for different scenarios. While industrially compostable plastics in post-consumer (bio)waste enable organic recycling, i.e., composting, for certain dedicated applications, many non-biodegradable bioplastics, especially drop-in solutions, can be seamlessly integrated into the circular economy to extend their lifetime through additional life cycles. In this perspective, the recycling of biopolymers is crucial to reducing the consumption of renewable resources needed for the synthesis of the corresponding monomers [216].

Four possible recycling pathways are available after bioplastic waste has been collected, sorted, and cleaned:

- Primary recycling allows us to recover polymers by mechanically recycling plastic waste, which has been pre-sorted by type and additionally by colour, application, etc., to obtain polymers of their original chemical structure and for similar applications [217]. However, this procedure currently only applies for PET (bottles).
- Secondary recycling is available for collected plastic waste fractions that cannot be ideally pre-sorted; however, this still allows the reuse of polymers in less demanding product applications in terms of their (thermo-)mechanical properties (downcycling) [218]. Currently, this procedure applies for most mechanically recycled materials.
- Tertiary recycling is performed through chemical and/or biological methods, depolymerising macromolecules into monomers or oligomers. The advantage of this method is its versatility and the possibility of delivering a pool of building block chemicals suitable for various applications [215,219].
- Quaternary recycling is the incineration of low-grade plastic waste for energy recovery.

In the context of a circular economy, priority should be given to the first three recycling methods whenever possible [217]. Still, the thermal recovery of biobased plastics contributes to a closed carbon loop by releasing the sequestered carbon dioxide back to the atmosphere from which it was taken up by the feedstock used in the beginning of the production process.

#### 4.2.1. Recycling and Repulping of Fibre-Based Packaging

Fibre-based packaging is currently recycled at a rate of 82% in Europe [47]. This is made via so-called repulping which allows the wastepaper to be converted back into pulp, which can be converted back into new items made from recycled paper. The potential for the defibrillation of paper- and paperboard-based packaging may be affected by the different coatings or additives used, and the recovered paper quality also depends on defects that can result from impurities in the pulp [220]. As such, there are different country-specific tests to assess the repulpability of fibre-based packaging. These country-realted specificities are due to differences in collection systems in the paper and board stream, among other factors. There are also different infrastructures (standard, deinking, specialized) throughout Europe [221].

In 4evergreen, a cross-industry alliance in which more than 85 entities of different sectors are collaborating to reach a 90% recycling rate by 2030, there is the endeavour to develop an assessment protocol for each main mill type to be endorsed in the value chain throughout Europe. However, every recycling mill technology somewhat differs. Standard mills are by far the most common type in Europe. Consequently, a draft of a harmonised testing method for these mills is already available [222].

In general, the recycling of paperboard involves several steps, such as pulping with detrashing, high-density cleaning, coarse screening with subsequent coarse reject handling, fine forward cleaning, fine slot screening with subsequent fine reject handling, light-weight cleaning, thickening with subsequent water clarification, and storage. Pulping with detrashing means to defibre the paper into its constituent fibres. While doing so, it is important to avoid significant degrading while the contaminants are being removed [223]. During these processes, less than 90% of the fed waste material can be recovered [224].

The recycling of beverage cartons is accomplished by specialized mills. In these mills, the plastic or polymer–aluminum fraction generally ends up in the mixed coarse rejects, if this fraction can be easily separated at the beginning of the repulping step. The mixed coarse rejects in turn are currently not further recycled for economic reasons [225]. These so-called repulping rejects are mainly valorised for energy or used in cements kilns as alternative fuels and bauxite substitutes [226]. Due to innovations in this field, the recyclability of beverage cartons has increased from 75% to at least 90%. In the course of this improvement, there have also been increasing non-food (packaging) applications in which the polymer–aluminum fraction is recycled into injected items [220,227].

#### 4.2.2. Recycling of Polymeric Monomaterials Using PLA as Example

With the exception of drop-in solutions, PLA is one of the most technologically advanced commercially available bio-based plastic materials. Therefore, the following subchapter will elaborate on the recycling of monomaterials using PLA as an example. Further information and literature on the recycling of the main conventional packaging plastics are provided by Schyns and Shaver (2020) [212], Thiounn and Smith (2020) [213], and Antonopoulos et al. (2021) [228].

The main recycling strategies exploited for PLA beyond industrial composting include mechanical and chemical routes, while enzymatic recycling is currently being considered as an additional EoL option.

Mechanical recycling allows the recovery of PLA, making use of well-handled and economically feasible industrial processes [229]. On the other hand, the thermo-mechanical processing of polymers in general causes a decrease in their chain lengths, and in the case of PLA, even its crystallinity can be affected, therefore making it necessary to monitor the number of possible recycling steps and the resulting quality of r-PLA [230]. Chain extenders are often introduced to restore molecular weight and improve mechanical properties [231].

Chemical recycling concerns the controlled degradation of the macromolecular structure of PLA to obtain high-purity products [232]. One approach consists of fully depolymerising the material to recover monomers by hydrolysis or alcoholysis. Hydrolysis at 160–180 °C for 2 h enables a 95% conversion of PLA to LA [233]. This method not only allows us to valorise post-consumer materials but also to produce LA in a more energy-efficient way than fermentation [234]. Similarly, alcoholysis can be applied for depolymerisation of PLA to obtain LA esters as value-added products [235]. In turn, lactide can be generated from these alkyl lactates, and can then be converted into PLA again, matching circular economy processes [236]. A different approach aims at partially degrading the macromolecular structure, obtaining oligomers for specific applications. A remarkable example is the fabrication of biobased adhesives deriving from partially depolymerised PLA [140].

Enzymatic recycling is developed as a new, promising, and sustainable approach for the treatment and re-utilization of polymers. Recycling polyesters by using hydrolytic enzymes can overcome the difficulties encountered with both chemical and mechanical recycling processes [237]. For example, the pyrolysis of PLLA requires high temperatures (approximately 250 °C), leading to the formation of DL-lactide monomers [238]. Conversely, recycling PLA by using enzymes occurs under mild conditions and does not determine the formation of undesirable by-products as racemic mixtures of PLA [239].

Several enzymes are used in a wide range of processes. Lipases, esterases, cutinases, and proteases families can catalyse the hydrolysis of different aliphatic polyesters, such as polybutylene succinate (PBS), PLA, PCL, PHA, and PPC [240,241]. Enzymes exhibit differences in substrate specificity and/or interfacial activation, as various types of hydrolases show remarkable differences in their three-dimensional conformation and their active site surrounding [242]. Furthermore, enzymes can hydrolyse polymers into monomers and/or low-molecular-weight oligomers from the chain-end (exo-type degradation), or along the main chain in a non-selective manner (endo-type degradation), resulting in high-molecular-weight oligomers as main degradation products [243,244].

The degradation of PLLA using commercial proteinase K from *Tritirachium album* was first reported by Williams (1981) [245]. Later, other serine proteases, such as α-chymotrypsin, trypsin, elastase, and subtilisin, have been shown to be capable of efficiently hydrolysing PLLA [246]. Kawai et al. (2011) [247] proposed two types of PLA-degrading enzymes with different enantioselectivities toward PLLA and PDLA: protease-type and lipase-type (including a cutinase-like enzyme), which preferentially hydrolyse PLLA and PDLA, respectively.

For over 10 years, studies of the enzymatic degradation of polymers into oligomers have been conducted with the main objective of reusing the products generated after enzymatic depolymerisation [248]. Kaihara et al. [249] found that lipase B from *Candida antarctica* was able not only to efficiently degrade PHAs but also to readily re-polymerise the obtained cyclic oligomers to produce the corresponding polyester. The biological recycling process of PLA was first reported by Youngpreda et al. [250] in which PLA powder was degraded by the protease produced by the *Actinomadura keratinilytica* strain T16-1 (approximately an 82% conversion) and the degradation products were then re-polymerised repeatedly by a commercial lipase.

Nevertheless, this promising approach still presents challenges to overcome. The enzymatic degradation of polymers with high crystallinity and high intermolecular force, such as PLA, takes a long time to be completed. Moreover, the enzyme and the degradation products form aggregates, which makes it difficult to recover and thus reuse the oligomers and/or monomers. Additionally, the enzymatic PLA-depolymerising method for the production of re-polymerisable oligomers requires a low amount of water in the system, leading to a low recovery yield of the oligomers [215]. Regardless of such challenges, the enzyme-catalysed transformation could open a promising route for the sustainable recycling of biopolymers.

#### 4.2.3. Multilayer Packaging Recycling

Multilayer plastic packaging materials are extensively employed to combine the respective properties of different polymers allowing tailor-made property profiles to be created with low material consumption. Since in many cases the polymer layers are usually thermodynamically immiscible, the recycling of multilayer packaging is an open issue; notably, plastic packaging is not designed for recyclability and, therefore, is largely incinerated or landfilled. Up to now, there have been two main methods to recycle multilayer items: one consists in the delamination of the system and the other one in the selective dissolution of the different components [4]. Other emerging recycling routes consist of compatibilization which allows for recycling in one stream and chemical processes, such as pyrolysis, that do not require the separation of layers [251].

To increase the recycling potential of post-consumer multilayer structures, the use of removable barrier layers in these structures is an emerging approach. For whey protein coatings, this has already been extensively proven at technology readiness level 4. In this case, during the recycling process, the washing stage has to be modified to allow the hydrolysis of the protein coating. Depending on the required process speed, this can be achieved by the use of enzymes or temperatures between 30 and 40 °C [53]. Although this recycling approach is specific to this specific biobased barrier coating, it requires very few changes and little investment for the recycling lines. These changes comprise modifying the sorting capabilities, if available, by tuning it with the right chemometrics package to detect the new materials, and optionally a density separation unit in case of multilayers composed of different types of plastic, e.g., PET/barrier/PE.

Moreover, the application of whey protein coating on biobased plastic substrates, such as cellophane of PLA, resulted in fully industrially compostable solutions with improved barrier properties [252]. Consequently, when combined with standard substrates, these multilayer solutions, which contain an intermediate layer based on whey protein, will benefit from improved material recyclability, otherwise when combined with biodegradable substrates, the multilayer structures will benefit from organic recyclability.

In the last few years, several Horizon 2020 projects have been funded by the European Union on this topic to find a circular solution to improve multilayer packaging recyclability. An overview of all these projects can be found via the Community Research and Development Information Service (CORDIS) of the European Commission. Among these projects, Terminus is adressing the challenge of enabling the reuse of flexible multilayer and multi-compound materials through delamination using a variety of polymers, which contain enzymesmediating intrinsic self-biodegradation properties, functioning as adhesives or tie layers. The technology is applied to biodegradable PU for adhesive and extrusion coating lamination and polymers, such as PBS or PLA, in blown extrusion. The aimed controlled biodegradation of either the adhesive or tie layer is supposed to enable the recovery of the different plastic layers, which can be then recycled using traditional methods. In any case, the use of innovative technologies involving enzymes can facilitate the degradation and recycling multilayer structures in the future.

### 4.3. Biodegradationg under Industrial and Home Composting Conditions

The first recycling process for which a testing strategy with specific requirements was developed was industrial compostability. This development led to the harmonized EU standard EN 13432, according to which a broad range of products, such as food containers, cups, sweets wrappers, and fill padding materials, is certified [253,254,255,256].

However, as already stated in Section 2, the exposed environment has an impact on a polymer’s biodegradation. This means that a given material that biodegrades by microbial activity under industrial composting conditions will not necessarily show the same or similar biodegradationunder home composting or aquatic conditions. [16] PLA, for instance, requires high temperatures (>50 °C) to start hydrolysis and biodegradation. These conditions are obtained during industrial composting processes, but are usually not reached during home composting. As a result, the biodegradation rate is slowed down or in some cases limited at lower temperatures. [16] Consequently, sufficient biodegradation and disintegration at ambient temperatures need to be proven in order to classify a material as home composatable. On a European level, no standard has been established yet for home-compostable products. However, since 2015, home compostability has become an important factor in France with the ban of conventional lightweight plastic shopping bags; bioplastics are encouraged to be compostable in home composting units and a French national standard on home-compostable materials has been introduced [257]. However, in line with the international and American specifications for industrial compostable products, it is likely that the European standard in discussion follows a similar approach. In that case, materials including small PLA amounts might not be able to achieve home compostability certification, as PLA is not home-compostable (see Section 2.1).

Test methods for determining the biodegradation of plastics under anaerobic conditions (ISO 15985) have been developed because biodegradation is influenced by the environmental characteristics. These characteristics include temperature, moisture content, pH value, oxygen presence, and microbial population. Although anaerobic digestion (AD) in biogas plants is considered organic recycling, according to the EU Packaging and Packaging Waste Directive, there is currently no standard existing for AD that defines specific requirements of biodegradation, disintegration, and possible effects on the digestate. However, EN 13432 already includes some general requirements for AD, as it is often combined with a composting step. AD has been under-investigated as an EoL option for bioplastics and packaging so far, but might gain more interest with the increased need to collect biowaste in the next few years. It is an interesting technology due to the additional biogas formation, which is suitable for electricity or heat production. In fact, the biobased and compostable plastics can have a positive effect on biogas production by contributing positively to the carbon/nitrogen ratio of the obtained compost or digestate [258].

Additional research has been undertaken to degrade PLA at mild conditions. Garrison et al. (2016) [259] reported the cleavage of PLA chains could be enhanced by the use of extracellular enzymes released by specific microorganisms, which contribute to the degradation processes. Recently, enzymes have become commercially available for enhancing PLA biodegradation in short-lived applications. It is used in PLA/PBAT-based films up to 60 μm thick [260]. Further research is being undertaken for home-compostable films, made of mainly PLA, with thicknesses up to 450 µm [261]. Moreover, the enzymatically enhanced biodegradation of PLA-based films in biogas plants could increase biogas production as well as improve AD performance and enzymatic activity in general [258].

### 4.4. Upcycling and Reprocessing

Mechanical recycling often leads to the downcycling of the recovered material, as already expounded in Section 4.2. This means that the recovered material that underwent downcycling during recycling can be reused but not in the same—high-quality—applications. Consequently, it will not contribute to lowering the demand for virgin plastic materials. Therefore, novel recycling strategies that result in the recovered material having the same quality as virgin materials or even superior properties, i.e., upcycling, are under investigation [262,263,264]. These strategies include a variety of different approaches, including the radiation of recovered materials and the reinforcement of said materials, which can be further subclassified in self-reinforcement and microfibrillated reinforcement. As both strategies represent innovative approaches to restoring the quality of mechanically recycled materials to the virgin level or higher, the following subchapters will explain these innovative approaches in more detail.

#### 4.4.1. Electron Radiation

E-beam has been tested as a novel food decontamination technology that uses low-dose ionizing radiation in the treatment of crops or food to eliminate microbial contamination. The ability of the electron beams to efficiently eliminate microorganisms is well known and their reliability inhigh-speed industrial production has been confirmed in numerous applications, particularly regarding the surface crosslinking of plastic [265]. However, the use for decontamination in packaging lines is muchmore recent. This progress is related, among other things, to the development of a new generation of emitters, which are more compact and durable. As summarised by Lung et al. [265], the environmental impact of decontamination with electron beams is very low, as neither water nor chemical products are required. 

As stated in Section 3.1.1, the irradiation of a given polymeric material can improve its mechanical and barrier properties by enhancing the crosslinking of the material. The extent of this increase depends on the polymeric material, the mix of polymers/fibres, and the applied radiation dose, as investigated by Manas et al. [266], who reported an increase by up to 36%. For PE/PA multilayer films, e-beam treatment at low doses (20–50 kGy) can enhance the barrier properties by up to 20% [267], whereas the mechanical properties in terms of tensile strength can be improved by up to 45% [268]. This is interesting considering that recycling of plastics in terms of material recovery may result in downcycling and the aggravated processing of said material.

E-beam treatment presents a potential approach to modifying the mechanical and barrier properties of recovered polymeric materials for upcycled secondary use. However, there is a risk that the use of e-beam treatment might result in tightly crosslinked polymer networks that are almost impossible to recycle in terms of remelting and can only be used as fillers. This in turn improves the mechanical properties of certain polymers as well [269,270,271,272]. Nevertheless, several studies have shown that e-beam treatments initiate crosslinking in r-PE—in some studies, even after multiple reprocessing events—and enhance mechanical properties [273,274,275,276].

In addition, the compatibility of polymer blends can also be improved by irradiation [277]. Polyester-based blends undergo a transesterification reaction, resulting in enhanced miscibility [278]. The electron irradiation of immiscible PE/EVA blends of different ratios was found to reinforce the interfacial interaction. In this case, irradiation did not evidently alter the blend morphology but rather stabilized it. This resulted in an increase in the tensile strength and a decrease in the elongation at break with increasing doses [279]. Besides polymer films, a recent study dealing with high-voltage cables reported that e-beam treatments showed promising results even for the compatibilization of ternary blends [280]. Moreover, Lazim and Samat [281,282] showed that irradiated r-PP can be used as a compatibilizer on microcrystalline cellulose reinforced r-PP composites to enhance the mechanical properties of said composites. Taken together, e-beam treatment presents a technology that can help to enhance the upcycling ability of various recovered polymeric materials. However, the effects of e-beam treatment on some (recycled) polymeric materials, such as r-PLA, bioPE, and bioPET, have not yet been investigated.

#### 4.4.2. Self-Reinforcement

Microfibrillar-reinforced composites consist of two phase materials. The reinforcing phase is embedded in a polymeric matrix phase. Usually, glass or carbon fibres are used for the reinforcement. The mechanical forces are directed from the matrix into these fibres. This allows for an improvement in the compatibility of the blend and a major increase in mechanical properties of the matrix phase. As a result, fibre-reinforced composites combine the advantage of high mechanical properties, in terms of strength and stiffness, with a low weight [262,283].

In self-reinforced polymer composites (SRPCs), the same polymer is used for the reinforcing and the matrix phase [284]. The concept was first introduced by Capiati and Porter [285]. SRPCs have both a high impact and high durability. However, their density is lower compared to that of traditional filled polymers. Using the same polymer type also eases the recyclability of such composites [286].

The result of self-reinforcing polymers is an increase in the following material properties: strength, stiffness, durability, and impact behaviour. The most significant improvement is observed for the impact behaviour [287]. SRPCs exhibit superior fibre–matrix adhesion. This is the result of the fibre and matrix consisting of the same material, as the high chemical similarity leads to a strong composite as well as to a high nucleation density for the fibres to transcrystallise into the matrix. As the fibre–matrix adhesion has a strong effect on the mechanical properties, poor fibre matrix adhesion leads to peeling, cracking, and reinforcement fibre pull-out [287].

Several polymers have been successfully used to manufacture self-reinforced composites, such as PP [288], PA [289], and PET [290].

Within this category of materials, biobased self-reinforced composites are defined as materials derived from renewable resources. Examples of self-reinforced composites studied so far include PLA, cellulose, nanocellulose, and starch. In this category, self-reinforced PLA composites, also named all-PLA composites, present the additional advantages of being compostable and of being one of the most studied materials with this feature [14]. Given the environmental challenges we are facing nowadays, composite materials that are derived from natural resources, biodegradable, and made of one single type of material represent an interesting alternative to traditional reinforced polymer composites.

In the BIO4SELF project SRPC, biobased prototypes based on PLA were demonstrated and these results are being commercialised. The potential of this approach is illustrated by BIO4SELF winning the 2019 Global Bioplastics Award [291].

The selection and preparation of PLA have an effect on the final properties of self-reinforced PLA composites. The selection of an amorphous or a semi-crystalline PLA grade is critical for the melting temperature, the crystallization rate, and the extent of crystallization. The processing of PLA into film or filaments also has an effect on the melting temperature and the mechanical properties of the film or filaments. To determine the most optimal processing conditions for self-reinforced PLA composites, it is essential to understand and characterize the effect of composite manufacturing conditions and crystallinity on the resulting mechanical properties of self-reinforced PLA composites.

#### 4.4.3. Microfibrillated Reinforcement

Immiscibility of the polymer constituents, the contamination of the polymer mixture by additives and fillers, and the interfacial separation in heterogeneous plastic waste are the main problems for mechanical recyclers. In general, the separation of polymer blends can be a challenge for the mechanical recycling industry. As a result, the reprocessing of contaminated blends at the final stage of mechanical recycling can result in poor mechanical properties due to the immiscibility of the polymer components. This is particularly true for blends of polar, e.g., PET, and non-polar, e.g., PP, plastics, making them difficult to reprocess into products for high-value applications [262].

However, as the incompatibility of the different polymers is a prerequisite for the application of the concept of microcrofibrillar composites (MFCs), this concept could possibly exploit the immiscibility of the polymer components to advantage for these types of mixed waste plastics. Developed by Evstatiev and Fakirov [292,293,294,295] in the early 1990s, MFCS are based on polymer matrix reinforcement with polymeric fibres. A more detailed description of the MFC process can be found in other publications [296,297,298,299,300,301,302].

Some research in the MFC field have already been carried out on recycled blends. The application of the MFC concept to the upcycling of r-PET bottels was demonstrated by Evstatiev et al. [303] with quite impressive results as r-PET reinforced virgin LDPE showed a tremendous increase not only in modulus and yield strength but also in impact strength. In addition, several experimental studies have been conducted on r-HDPE and r-PET MFCs. Lei et al. (2009) [304,305] successfully processed r-HDPE and r-PET MFCs by adding various compatibilizers, resulting in a significant increase in toughness for MFCs containing 5% (weight percentage) of ethylene-glycidyl methacrylate. Furthermore, Jiang et al. [306] found that both HDPE and PET components showed some degree of photo-degradation in a study on the effect of UV exposure on HDPE/PET MFCs. However, in situ MFC processing processing improved the mechanical and thermal properties of photodegraded polymers, and the yield strength increased with increasing UV exposure time [306]. In a study on MFC recyclability, Jiang et al. [307] found that the tensile strength increased with the number of re-processing steps. Kuzmanović et al. [308], on the other hand, used mixed plastic waste as a matrix in the production of MFCs to try upcycling plastic waste. Despite the limited number of matrices currently tested, the MFC concept has proven to be applicable for the upcycling of mechanically recycled polymeric materials. A new type of recycled fibre-reinforced composite could result from the combination of both approaches—the MFC concept and compatibilization—for the upcycling of polymer waste.

## 5. Safety Environmental, Social, and Economic Impacts

### 5.1. Assessing Environmental, Economic, and Social Impacts of Biobased Packaging

**Figure 4:**
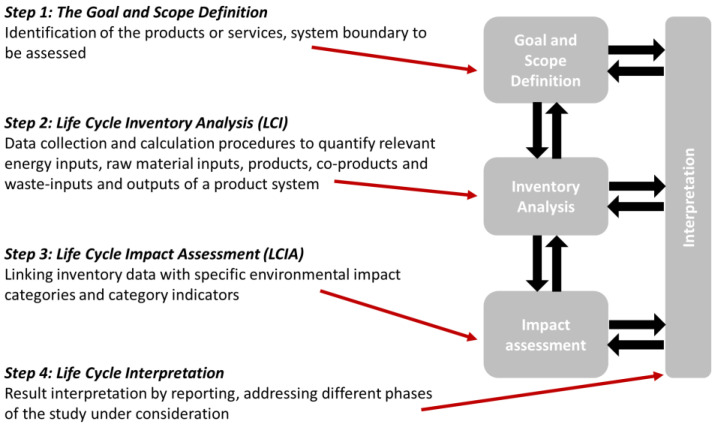
Illustration of the general phases of a life cycle assessment according to ISO 14040:2006.

A life cycle assessment (LCA) is a well-established tool to shed light on the environmental performance of products, such as biobased plastics, and production systems by quantifying their cradle-to-grave (or gate) contribution to a range of impact categories (ISO 14040:2006 and ISO 14044:2006). Therefore, LCA aims to quantify all the inputs and outputs of material flows and to assess how these material flows affect the environment in order to compare the full range of environmental effects attributable to products and services. As depicted in Figure 4, this information can be used for process improvement, policy support and informed decision-making.

The European Commission has been promoting the concept of the biobased economy since publishing its first strategy for “Innovating for Sustainable Growth: A Bioeconomy for Europe” in 2012, which contains a general definition of the “bioeconomy” and an overview of its features [309]. This strategy document states that the bioeconomy “encompasses the production of renewable biological resources and the conversion of these resources and waste streams into value added products, such as food, feed, biobased products and bioenergy, includes the sectors of agriculture, forestry, fisheries, food and pulp and paper production, as well as parts of chemical, biotechnological and energy industries” [309].

Similar to fossil-based materials, biobased plastic materials and processes can have intended and/or unintended impacts on the environment and human society. These impacts may occur throughout the value chain. As each packaging material has unique properties, the life cycle of the respective material is also unique. Consequently, the aforementioned impacts can apply to different indicators and can be linked to the actual characteristics and effects of the new, biobased products and to different stages. These stages may include, among others, the production of biomass, biorefinery and related processes [310,311]. Notably, a single product or process can have multiple impacts. These impacts are also influenced by external factors that may be specific to the product or process in question. Consequently, these impacts are context-specific and can be partly positive and partly negative. This in turn means that stating whether the overall impact of a product or process is negative or positive remains challenging [310,311]. This is especially true when attempting to compare LCA data derived from distinct studies, as summarized by Bishop et al. [312] and Spierling et al. [313].

#### 5.1.1. Environmental Impacts

**Figure 5:**
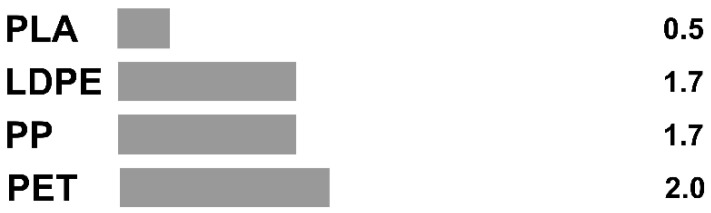
CO_2_ equivalent emissions in kg per kg of polymer—cradle to gate. Adapted from BioApply.

**Figure 6:**
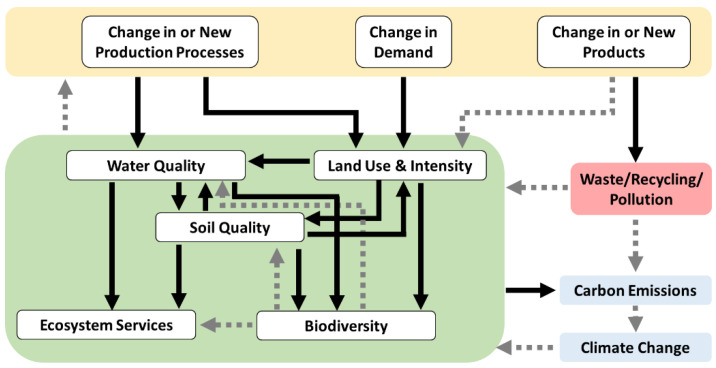
Overview of environmental impacts to be taken into account (black arrows), and indirect interdependencies (grey doted arrows) that excessively complicate environmental LCA. Adapted from [311,314,315,316].

The promotion of bio-based plastics and other bio-based products could have beneficial environmental impacts on the bioeconomy as it simultaneously drives the replacement of petroleum or other fossil-based products. This substitution, in turn, not only encourages independence from finite resources and a reduction in import dependency, but also promotes the reduction in CO_2_ emissions during production, as depicted in Figure 5 [311,314]. However, carbon emissions are not the only environmental impacts to be taken into account. As depicted in Figure 6, changes in demand production processes or products as well as the introduction of new products and production processes impact the environment and the local ecosystems in terms of several aspects, such as land use/intensity and soil and water quality [311,314,315,316]. These impacts, in turn, affect biodiversity and ecosystem services, which again can influence inter alia water and soil quality and so on. These interdepencies excessively complicate the assessment of environmental impacts.

#### 5.1.2. Social Impacts

Assessing the social impacts of (new) markets and production processes, despite growing scientific interest, has not yet been standardized or even generally defined within the scientific community. Therefore, to calculate potential effects on employment, health, and food security, further methodical improvements and data collection are needed, a procedure analogous to the development of the (more advanced) life cycle assessment as defined by ISO 14040/44. Current methodologies for assessing social impacts at the product level are not only immature. They also need to be flexile, as certain requirements depend on the specific background of a product. As summarized by Reinales et al. [317], the stakeholders need to be addressed and consequently the corresponding categories and indicators that allow social impact assessments have to be identified according to the relevance of the new product or process. The drivers of social impacts may have an economic background. This means that they refer to questions about the distribution of income, economic possibilities and access to land, markets, seed capital, and technology [311]. For example, communities or individuals who do not benefit from the bioeconomy can potentially be identified based on these access limitations. Reducing the variety of indicators to a final indicator of quality of life, people’s quality of life will be impacted by access issues equally as by changes in food security, employment, household income, health status, or food prices [311]. However, as mentioned before, assessing social impacts has not yet been standardized and therefore, the applied methodologies need to be selected in accordance to the product or process to be assessed.

#### 5.1.3. Economic Impacts

Assessing the economic impacts of a new product or process is crucial to addressing the economic viability of said product or process, although this has not been standardized yet [313]. As summarized by Hasenheit et al. [311], technological innovation is a key driver. Nevertheless, other impact indicators, such as the changing demand for bioeconomy-related feedstock (=input) and products, must not be neglected. In this case, an increasing demand (for bioeconomy-related feedstock and product) may lead to a decrease in the demand for fossil fuel-based products, depending on the extent to which the said feedstock production depends on fossil fuel [311]. Moreover, the aforementioned increasing demand for said feedstock and products can lead to changes in the price of the corresponding sources and commodities, which in turn may put pressure on other consumers of these commodities [311]. In contrast, due to increased demand for feedstock and input for the bioeconomy, the producers of the aforementioned commodities may obtain significant economic perspectives in terms of new sources of income. On the other hand, production methods, biomass productivity, and processing may experience changes related to new bioeconomy-related processes. Overall, regional and national trade balances could be significantly influenced by changing demand and prices for bioeconomy-related products and processes. In this case, the overall gross domestic product and gross national income may be affected by new markets and changing trade balances [311]. Nevertheless, it should be noted that the economic impacts may be affected by environmental impacts and may in turn influence social impacts [313].

### 5.2. Safety Assessment

Over the last two decades, society has become increasingly aware of the environmental risks posed by plastic pollution, as it enters the environment in an uncontrolled, increasing and undeniable manner [318].

The new Packaging and Packaging Waste Directive (EU) 2018/852 [319] aims to promote eco-design of packaging to achieve high-quality EoL management of plastic waste. Responsible and risk-free consumer behaviour may be promoted through an eco-design approach for plastic products such as packaging. Ensuring safety in the plastics sector goes beyond trying to make plastics more sustainable, to making their use more environmentally friendly, and trying to minimise the use of foil-based plastics [320]. It is also about ensuring their safe use for the workers who work with them during manufacturing and waste management, for instance. Moreover, it is crucial to ensure that there is zero risk when plastics come into contact with food. The same is true for trying to prevent microplastics from reaching aquatic environments, where they are available to living organisms that ingest them and pass them into the food chain [320].

In contrast to plastics intended for contact with foodstuffs or cosmetic packaging, there is no separate regulation for the safety assessment of plastics in general. Therefore, the safety assessment of bioplastics has to follow the same guidelines as conventional plastics. This involves identifying all the risks associated with their use, including professional, consumer, EoL or reuse, and management as waste, and defining the different scenarios for which a potentially harmful effect, both human and environmental, may occur. From the point of view of human health, it is essential to study the potential toxicological profile following OECD guidelines [321], as well as the possible routes of entry into the human body, in order to establish the necessary corrective measures to guarantee their safe use. From the point of view of environmental health, it is essential to know the possible routes of entry into different ecosystems to ensure their biodegradation potential by means of the standardised norms described above and to make sure that, during the time they are present in the environment, they do not cause harmful effects on organisms.

#### 5.2.1. Environmental Safety Assessment

In aquatic environments, particularly in the marine environment, theharmful effects of plastic pollution are clearly visible [322]. Marine flora and fauna are particularly harmed by so-called single-use plastics, such as plastic bags and straws [323]. Nevertheless, many people remain unaware of the laws and regulations interdicting the disposal of plastic into marine environments [324]. There are a number of examples of ongoing international legislative actions to address the problem of plastic pollution in the marine environment resulting from plastic bags, other single-use plastics, and microbeads. This action has been supported by an increase in public awareness driven by international organisations [323].

One of the negative effects of plastic that has recently been attracting attention is the presence of man-made microplastics in aquatic environments, as they are now known to have significant adverse effects, directly or indirectly, on terrestrial and marine wildlife, as well as human health [325,326].

As public awareness increases, it is important that environmental legislation to support the mitigation and control of plastic waste in marine environments, so that in the future the disposal of plastics in the sea may be prevented [327]. In some European countries, growing public awareness has already helped to tax or bans single-use plastics [328]. Biobased plastics can significantly contribute to increasing resource efficiency through a closed material cycles and cascade use, especially when biobased materials and products are reused, recycled, or used for energy recovery.

#### 5.2.2. Human Safety Assessment

In the assessment and improvement of the environmental performance of bioplastics and plastic alternatives, the focus is either on the production phase, e.g., the carbon footprint and renewable feedstocks, or the EoL phase, e.g., industrial compostability. When assessing the sustainability of materials made from fossil-based plastics and biobased plastics, the performance during use phase, such as human exposure to chemicals, is often not adequately considered [329,330]. The chemical safety of biobased plastics, i.e., the identity of the compounds contained in the material and their (mixture) toxicity as well as human exposure to these compounds, needs to be assessed in the same way as for fossil-based plastics. This is important as human exposure to chemicals contained in bioplastics and plant-based materials will increase as their use increases.

#### 5.2.3. Food Packaging

Food comes into contact with many materials and articles from production, to consumption. These materials and articles are known as food contact materials (FCM). Examples of FCM are containers for transporting food, processing machinery, packaging materials, or kitchenware and tableware [331].

With the establishment of the Single Market in 1993, the European Economic Community has set up an area where, among other things, the free exchange of goods, services, and capital is assured, as further specified and supported by the Single Market Act [332,333].

In the sector of materials and articles intended to come into contact with foodstuffs, the legislative work needed to implement the necessary harmonisation focuses on two essential goals:

- The removal of the technical barriers to trade;
- The protection of the health of consumers

Taking into account these two essential goals regarding trade and the protection of health, FCM legislation has established a harmonised legal EU framework, by means of the Regulation (EC) No. 1935/2004 [334], on materials and articles intended to come in contact with food, from which the rest of the applicable regulations are structured. The framework on the regulation of food sets out the general principles of safety and inertness for all FCMs. The general requirements of this Regulation stipulatethat these materials and articles must be manufactured in accordance with good manufacturing practice (GMP) to guarantee that, under normal or foreseeable conditions of use, their constituents are not transferred to food in quantities that could:

- Endanger human health;
- Bring about an unacceptable change in the composition of the food; or
- Bring about a deterioration in the organoleptic characteristics.

This Regulation requires the European Commission to establish specific measures for different materials, by means of directives or regulations. Annex I of Regulation (EC) No. 1935/2004 lists 17 groups of materials and articles, which may be subject to specific measures. So far, there are only specific measures for five types of materials: plastics, recycled plastics, active and intelligent plastics, ceramics, and regenerated cellulose. When specific measures are not available for a given material, it is common to rely on national legislation in Member States, Council of Europe resolutions, or recommendations adopted by different institutions and organisations to help industries meet the food safety requirements set out in the Framework Regulation. In the case of plastics, Commission Regulation (EU) No. 10/2011 and the corresponding amendments describe the limit values for the migration of monomers used for polymer-based materials intended to come in contact with food. Most monomers are given a specific migration limit, which is expressed as milligrams per kilogram of food (mg/kg). If no specification exists for a material, an overall migration limit of 60 mg/kg applies [335].

One of the four basic requirements of the Framework Regulation is the application of GMP for the production of food contact materials. To ensure a harmonised application of GMP across the EU and in the different links of the supply chain, the basic principles of GMP are laid down in Regulation (EC) No. 2023/2006 [336]. This regulation applies to all sectors and all stages of the manufacturing, processing, and distribution of materials and articles, but excluding the production of starting substances. For example, for the production of plastic, GMP requirements start with the plastic manufacturer followed by the converter, including the printing process of the packaging material and up to the production of the finished article.

Together with Regulation (EC) No 1935/2004, Regulation (EC) No 2023/2006 on good manufacturing practice for materials and articles intended to come into contact with food is crosscutting and mandatory for the entire food packaging industry, irrespective of the material used and the food product packaged. Again, all mentioned regulations are relevant for both fossil-based and biobased FCM.

In summary, every FCM in Europe must comply with the following Regulations:

- Food Contact Materials Framework Regulation;
- Good Manufacturing Practice Regulation;
- Specific measures: harmonised regulations/legislative texts/recognised recommendations or guides, etc., which are applicable to the specific type of material;
- Other legislative texts and supporting documents.

Therefore, in-depth studies on the applicable legal framework for the respective packaging solutions are required.

#### 5.2.4. Personal Care Packaging

According to Regulation (EC) No. 1223/2009 on cosmetic products [337], a cosmetic is any substance or mixture intended to be placed in contact with the external parts of the human body, such as epidermis, hair system, nails, lips and external genital organs, or with the teeth and the mucous membranes of the oral cavity with the aim exclusively or mainly to clean, perfume, or protect them, as well as change their appearance, keep them in good condition, or correct body odour.

In accordance this Regulation (EC) No. 1223/2009 concerning cosmetic products, cosmetic products placed on the market must be safe for human health when used as intended or under reasonably foreseeable conditions. To fulfill this requirement, the person responsible for placing the product on the market has to assess its safety based on the intended use of the cosmetic product and the expected exposure to each component.

Therefore, for the legally required safety assessment of the final cosmetic product by the safety assessor of the responsible person, the documentation provided by the packaging supplier is an important building block. Several industry organisations, representing the value chain of cosmetic packaging, have been collaborating to develop a common understanding of the appropriate packaging material data to provide to cosmetic product safety assessors, as there are no detailed regulatory requirements for the exchange of information along the supply chain. In this sense, an approach has been given involving the following principles to be observed:

- Requirements of the Cosmetics Regulation;
- Requirements regarding REACH, the Packaging and Packaging Waste Directive 94/62/EC and other legislation;
- Requirements of FCM legislation in Europe.

If not stated otherwise, the aforementioned regulations are relevant for fossil-based and biobased materials. Moreover, also in the case of personal care packaging, in-depth studies on the applicable legal framework for respective packaging solutions are required.

## 6. Industrial Applications

In regards to product application and development for bioplastics, packaging remains the largest field of application for bioplastics with 47% of the total bioplastics market in 2020, which equates to 0.99 million tonnes. Nevertheless, the use of bioplastics is expanding into a wider range of markest, including catering products, consumer electronics, toys, textiles, and agriculture/horticulture, among others. Additionally, industries such as building and construction, automotive and transport, and electric and electronics are experiencing an upward trend, fueled bythe increasing capacities of functional polymers [12].

According to Research and Markets [338], rigid bioplastics applications will hold a dominant position in the market, especially for cosmetics applications, such as compact powders, lipsticks, creams, and beverage bottles. The market leaders in those fields already use biobased PET or PE for their packaging needs. PLA is also gaining a greater market share for rigid packaging [338]. Other market leaders in Europe have already introduced bioplastics into the market. As a rising number of big brands resort to bioplastic solutions, consumers will adapt and increasingly accept these products. With growing demand and increasing volumes of bioplastics on the market, production costs will soon adapt to the prices paid for conventional materials [12].

In the following subchapters, only a few selected examples of the possible industrial applications of secondary raw materials (SRM) and of biobased plastics are given. Further information can be found as summarized by Detzel et al. [339] and Nilsen-Nygaard et al. [340].

### 6.1. Industrial Application of Biobased Food Packaging

The intent to provide packaging solutions that are more sustainable than well-established (fossil-derived) packaging products does not stop at packaging manufacturers. Customers and legislators are also increasingly taking interest in this subject. Therefore, it is not surprising that numerous products have recently been launched on the market representing either completely biobased and/or compostable food packaging solutions. Moreover, the recyclability of the materials is an important key to close the loop and ensure the sustainability of these packaging solutions.

An example for the latter case was launched by a chemical company and a company active in forest and bioindustry. They represented wood-based renewable naphtha that can be used to develop plastics, such as biobased PE [341]. The resulting polymeric material can then be applied for instance in coffee packaging or as label film [342,343].

To reach more sustainability, companies do not risk everything on one endeavour; instead, they take different approaches. A food, snack, and beverage corporation and a biopolymer manufacturer developed a “thin-film plant-based snack packaging” based on PHA [344]. However, the industrial application of solutions based on PHA is a big challenge, because of its limited availability and current price. The production costs of biodegradable plastics, such as PHA, are 20% to 80% higher than those of conventional plastics [345]. Furthermore, the same food, snack, and beverage corporation joined a consortium of global consumer goods companies to further develop a recyclable paper-based bottle produced by a packaging technology company [346]. According to the involved packaging technology company, the bottles are PET-free, but “specialized coatings” are applied for the bottles to be compatible with their contents. Other examples of paper-based bottles have a thin biobased polymer film that acts as a barrier [347,348]. Furthermore, approaches designed for composting by combining fibre-based solutions with PLA films or designed for recycling by combining fibre-based solutions with barrier liners are increasingly used in food packaging and food service sectors to replace fully fossil-derived solutions and match the demand for plastic reduction [349,350]. Indeed, the use of fibre-based packaging solutions is preferred by consumers, because they perceive these materials as more environmentally friendly [351].

Biodegradable and biobased materials are gaining popularity every day and are used by many converters for packaging solutions. For example, in 2020, a packaging manufacturer and a chemical company developed a home-compostable three-layer cling film based on PBAT that is suitable for packaging various products [352]. In 2021, another packaging manufacturer in cooperation with a mechanical engineering company developed composability-certified biobased packaging for vegetarian and vegan products using a biodegradable film from the aforementioned chemical company [353]. Moreover, protein-based films and coatings are hot topics. In 2013, a German company specializing in the extraction and processing of milk protein filed a patent related to a method for producing milk-protein-based plastic material [354] which resulted in the production of milk-protein-based films [355]. Moreover, a British meal kit retailer teamed with a mission-based commercial spinout of a British university and in 2021 launched an edible film made of pea protein that is specifically engineered for a commercial stock cube packaging application as it dissolves in hot water [356]. Although this specific film is claimed to be the world’s first edible packaging solution made from pea protein, it should be noted that the application of pea protein in (edible) films has been under investigation for more than two decades [357,358].

In contrast to most approaches described in this section, which mainly pursue the use of virgin biobased materials, there is also another important focus to improve the sustainability of packing solutions. The use of recycled materials for food contact packaging has taken an important role in the current market. In 2019, two chemical companies together with a packaging manufacturer and a dairy company developed prototypes for mozzarella packaging using 100% recycled materials (Ntemiris, 2019). Furthermore, in 2020, the same packaging manufacturer and a meat and meat products manufacturer again together with two chemical companies developed multi-layer packaging for organic poultry sausages, in which more than half of the packaging materials were recycled [359]. In both cases, chemical recycling was applied, and the recycled material was distributed by a mass balance approach. The combination of these innovations may open new opportunities to extend the inclusion of recycled and biobased packaging solutions.

### 6.2. Industrial Application of SRM for Textiles, Composites, and Personal Care Packaging

The clothing industry is suffering from the high ratio of blends of synthetic and natural fibres. Both technical textiles/clothing companies are looking for materials that can undergo decomposition in landfill conditions, are compostable, or are easily biodegradable. The synthetic material, furthermore, must be able to undergo the load of the standard life cycle of the fabric [360]. Therefore, the most common synthetic materials are viscose, PA, polyester, PU, and polyacrylonitrile [361]. In technical textiles, the share of natural fibres is lower, as they are defined as technical fibres, materials, and support materials meeting technical rather than aesthetic criteria. Consequently, these kinds of textiles can be utilised in various applications, such as automotive, agriculture, road construction, medical and hygiene products, packaging, and personal protection [362]. When used as blends of different materials, e.g., synthetic and natural fibres, the separation and sorting of the different types of fibres is rather complex, resulting in low streams of recycled polymers [363].

In nonwoven textiles, the use of recycled polyester is well established. In 2017, polyester comprised 30% of the raw material used in nonwoven textiles. This share was composed of 40% recycled polyester [364]. More importantly, the use of recycled polyester in nonwoven textiles is expected to increase from 34% in 2017 to 40% in 2025 [364]. The biggest use of nonwoven textiles is for hygiene; PP and PE are the key players. More than 25% of all nonwoven textiles produced in the EU are meant for hygiene applications [365]. These include, for example, wipes, single-use diapers, and adult incontinence products.

In the field of packaging for personal care, fossil-based materials are mainly used. In order to be more sustainable and support a circular economy, some manufacturers actively work on finding and connecting to other sustainable sources. One option to contribute to sustainability is changing the source of the material from fossil-based to recycled materials, such as post-consumer recycled (PCR) content, ocean waste materials, or biobased materials. Another option is weight reduction, as this also lowers the carbon footprint of packaging. As the demand for sustainable materials is high, the most frequent feedback is that materials are unavailable sometimes up to a year in advance. This presents another challenge when developing new products and working with recycled or biobased materials. As experienced and personally communicated by Romei SRL in February 2021, in Italy, the use of post-industrial r-PLA is held back by two factors. One factor is the lack of processing plants optimized for PLA recovery, which results in the potentially poor processability of r-PLA. The second factor is the uncertain product life span. Nevertheless, upon blending with PET or PE, r-PLA has been accepted by customers, as it has shown a good performance in the extrusion of monofilaments. This statement is further supported by several studies demonstrating that low percentages of (recycled) PLA are miscible with r-PET and certain recycled polyolefins, and do not negatively affect the performance of these materials [366,367,368,369].

Currently, one challenge when using PCR is monetary. Virgin material is about 20 to 30% cheaper than recycled material of similar quality. Moreover, depending on the plastic, the availability of recycled material is often limited [370]. On the other hand, due to the regulatory framework, the use of PCR in personal care packaging is facilitated compared to that of food packaging. Despite the challenges that may occur with the application of PCR, several companies successfully use recycled materials in personal care packaging. Multiple companies already use PCR materials on caps for cosmetic packaging or also directly in cosmetic packaging [371,372].

Shower gel bottles produced by a manufacturer of cleaning and care agents are another example of the application of recycled materials for personal care packaging. In this case, this particular cleaning and care agent manufacturer partnered with a manufacturer of plastic resin and an engineering recycling company to develop the first cosmetic packaging that is based on 100% PCR HDPE. The used PCR material has even been approved as food safe by the FDA [373,374].

## 7. Challenges and Future Prospect

Over 368 million tonnes of plastic are produced annually and less than 1% of this share is formed by bioplastics. Yet, the demand is rising, and global bioplastics’ production capacity is set to increase from around 2.42 million tonnes in 2021 to approximately 7.59 million tonnes in 2026, which represents an increase of 200% in 5 years [1]. The increasing attention towards more sustainable products is continuously stimulating the production of bioplastics applications.

The bioplastics packaging market was valued at USD 14.85 billion in 2019 and is expected to reach USD 39.37 billion by 2025 [375].

### 7.1. Market Trends

Contrary to a slight decrease in the overall global plastic production in 2019 and 2020 due to the coronavirus pandemic, the market for bioplastics has continuously grown. The driving force behind this development is growing demand coupled with the development of more sophisticated applications and products. According to the latest market data compiled by European Bioplastics in cooperation with the nova-Institute, global bioplastics production capacities are forecasted to triple in the next five years, and the share of bioplastics in global plastic production will pass the 2% mark for the first time [1]. However, this positive trend will be heavily dependent on the price of conventional plastics, along with other factors, such as technological progress, economies of scale, raw material costs, and policies to promote sustainable alternatives to fossil-based plastics [376].

All these macroeconomic factors (price of crude oil, GDP growth, and feedstock price), regulations (taxes, subsidies, bans), technical and technological factors (scale effects, learning rates, economies of scale), and social effects (consumers awareness) are subject to sudden or unforeseen changes and can vary greatly. Consequently, it has to be emphasized that real market trends beyond 2025/26 are difficult to predict [376].

### 7.2. Market Trends and COVID-19

In general, trend analyses have been carried out considering a steady increase in both oil prices and GDP. However, the COVID-19 crisis led to a massive decrease in oil prices between −50% and −85% [377] as well as a decrease in the GDP around the world of −1% [378]. Moreover, the COVID-19 crisis has influenced and will continue to influence the foreseeable market dynamics of biodegradable bio-based plastics until 2030 [376]. The European Union reacted with a Recovery Plan for Europe [379] of €1.8 trillion, with €143.4 billion dedicated to innovation. In addition, the need for health-safe packaging and the massive demand for personal protective equipment, such as face masks or gloves, may have caused a shift in social awareness concerning the use of plastics [380]. Evidently, COVID-19 had a great impact on three of the factors that can influence market trends for plastics, namely macroeconomic factors, regulations, and social effects. Official studies about the real effects on the demand for bioplastics after COVID-19 have not been delivered yet. However, European Bioplastics observed that the industry did not suffer any shift or change during 2020, which leads to the impression that the market trend for bioplastics will not suffer from the COVID-19 crisis [12].

### 7.3. Economy and Job Growth

The economic potential of the bioplastics industry also reflects on the number of jobs related to the field. According to a job market analysis conducted by EuropaBio, in 2013, the bioplastics industry accounted for around 23,000 jobs in Europe, while the forecast predicts that up to 300,000 highly skilled jobs will be created in the European bioplastics sector by 2030 [381,382]. Of course, this does not take into account the impact of COVID-19 on the job sector of bioplastics.

### 7.4. Regional Share of Bioplastic Production

With a view to regional capacity development, Asia further expanded its position as a major production hub in the past few years with almost 50% of bioplastics currently being produced in this region. It is predicted that over 70% of bioplastics will be produced in Asia by 2026 [1]. The increasing awareness among Asian consumers, and the strict ban of conventional single-use plastic by China, India, and Japan increased the consumption of bioplastics in the region [375].

Currently, almost a quarter (24.1%) of the production capacity is still located in Europe [383]. However, Europe’s share, as well as the share of other regions will decline significantly ithin the next five years. It is predicted that by 2026, only around 17% of bioplastics production capacity will be located in Europe [384]. Nevertheless, demand for bioplastics will continue to be led by Germany, the largest economy in Europe and the fifth largest in the world, followed by France, Italy and the United Kingdom, the latter being the fourth-largest consumer of plastics in Europe [375].

As stated by European Bioplastics (2020) [12], Europe could play a central role in the development of the share of bioplastics use. Europe offers excellent conditions to compete globally for future markets and technologies, thanks to several factors, such as leading global companies both in the chemical and plastics industries as well as in industrial users of plastics, and a highly aware society with strong purchasing power.

### 7.5. Land Use

The land used to grow the renewable feedstock for bioplastics was estimated at approximately 0.7 million hectares in 2021. This represents roughly 0.015% of the global agricultural area of 5 billion hectares—of which 94% was used for pasture, feed, and food [1]. According to European Bioplastics and Nova Institute (2021) [1], the market growth predicted for bioplastics is not going to affect the use of land to produce feedstock. In fact, in the next five years, the estimated share of land use for bioplastics will only slightly increase to below 0.06%. This shows that there is no conflict between the use of renewable feedstock for food, feed, and the production of bioplastics [1].

### 7.6. Challenges

One of the main challenges is represented by the high price of bioplastics production: many bioplastic materials significantly exceed the costs of the fossil-based plastics used for the same or similar applications, although in some cases price competitiveness is in sight [385].

Other challenges result from the need of transparent and clear communication in the complex field of biobased products. In fact, it is not always clear to general consumers that for example “biobased” does not necessarily mean that a product is “biodegradable”. Additionally, consumers need clearer data on how much biobased content is in the purchased packaging, how much CO_2_ emissions are saved, and if the biomass used to produce bioplastics was grown sustainably [376,386].

A big challenge is also the missing level playing field for biobased plastics compared with fossil-based plastics. Despite their small market share, biobased plastics are often seen very critically regarding their actual environmental impact and sustainability contribution. Whereas it makes sense to thoroughly review new materials for potential advantages and disadvantages, the same measures and demands are not applied to established fossil-based plastics, which is only cementing their already dominating position in the market and unnecessarily raising the hurdles for new innovative technologies, such as bioplastics.

Furthermore, unlike policy support for biofuels and renewable energy, no EU-wide legislative framework currently exits to promote the use of renewable raw materials for plastics solutions. The US and Thailand have installed a supporting policy framework, which has led to the increased use of these materials in the corresponding markets [387,388].

### 7.7. Regulatory Framework

The bioplastics industry is affected by European regulatory frameworks and specific policy measures, albeit often more indirectly. For example, legislation and policies that focus on the recyclability of and reduction in plastics have an indirect effect on bioplastics. When the Commission issued the goal of making plastics 100% recyclable or reusable by 2030, but not considering the benefits of and setting specific targets for the market presence for bioplastics [376], it was missing a chance to promote the use of bioplastics to help make the plastics industry more sustainable.

At the same time, there are several legal acts and European strategies that directly support the development of bioplastics. The EU Action Plan for the Circular Economy [389] and its update [390] promote the “efficient use of biobased resources through a series of measures, including guidance and dissemination of best practices on the cascading use of biomass and support for innovation in the bioeconomy”. Furthermore, the “revised legislative proposals on waste contains a target for recycling wood packaging and a provision to ensure the separate collection of biowaste” [389].

The Roadmap to a “Resource Efficient Europe” [391] aims to tackle the objective of making Europe resource-efficient by supporting research on biodegradable plastic. The “Bioeconomy Strategy” [392] aims to foster biobased products and identifies the bioeconomy as essential in developing alternatives to fossil-based materials that are also marine-biodegradable.

The EU Strategy for Plastics, adopted in 2018, suggests actions to enhance efficiency of the European plastics system’s use of resource. The objective is to ensure that all plastic packaging on the EU market can be reusedor recycled by the year 2030. The strategy also initiated a ban onf selected single-use plastics. Concerning biobased and biodegradable plastics, the Commission underlined the importance of clear communication to consumers on how to use and dispose biodegradable plastics [184].

In EU Directive 2018/851, EU Member States are encouraged to promote an increase in the share of reusable packaging. In addition, it acknowledges that fostering “a sustainable bio-economy can contribute to decreasing the Union’s dependence on imported raw materials. Biobased recyclable products and compostable biodegradable products could represent therefore an opportunity to stimulate further research and innovation and to substitute fossil fuel-based feedstock with renewable resources” [393]. This directive amended the Waste Framework Directive 2008/98/EC [394].

The EU Directive 2019/904 “on the reduction in the impact of certain plastic products on the environment” requires all plastic packaging be either reusable or recyclable, by 2030 [395]. In addition, this directive bans the placing on the market of selected single-use plastic products, such as straws, cutlery, and plates since June 2021, encourages the reduction of other single-use items, such as cups and food containers, and obliges producers to inform consumers about the negative impact of plastic waste [395]. As regards the implementation of this Directive in the Member States, it seems very likely that grave problems will result from it. There are currently pending guidelines that are intended to provide answers to questions as to what exactly defines a single-use plastic product and when a polymer is deemed to be regarded as natural, chemically unmodified and, therefore, outside of the scope of the Directive. These guidelines will inevitably lead to differences in national legislation incompatible with the functioning of the internal market.

Directive 1999/31/EC on the landfill of waste [396] obliged Member States to “set up a national strategy for the implementation of the reduction of biodegradable waste going to landfills (…)” and to ensure a stepwise reduction in “biodegradable municipal waste going to landfills (…) to 35% of the total amount (by weight) of biodegradable municipal waste produced in 1995…”, 15 years after the deadline for the implementation of the Directive into national law.

Directive 94/62/EC on “Packaging and Packaging Waste” [397] directly contributes to preventing or reducing the impact of packaging and packaging waste on the environment and to ensuring the functioning of the internal market. The directive also contains provisions on reusing packaging and on the preventing, recovering and recycling of packaging waste. The amount of packaging waste to be dealt with is growing. Therefore, the European Commission is currently assessing the “Essential Requirements” that all packaging intended to be brought onto the market must fulfil. The aim of the current assessment is to make all packaging more rigid and enforceable, in order to reduce packaging waste.

Furthermore, the “7th Environment Action Programme” [398] advocates for additional efforts in transforming the EU into a resource-efficient, low-carbon economy. It includes a special focus on more prevention, reuse, and recycling. 

The objectives of the “Eco-innovation Action” [399] focus on boosting innovation that results in or aims at reducing pressures on the environment and on bridging the gap between innovation and the market. The “European Environmental Technology Action Plan” [400] intends to develop eco-efficient ways to convert biobased raw materials and waste into biobased plastic products and to support the development of eco-technologies promoting foreign investments in environmental technologies, leading to increased employment and economic growth in the EU.

Additionally, priorities indicated in the “European recycling society” [401] present the long-term vision of the thematic strategy on the prevention and recycling of waste.

The “European Lead Market Initiative” for biobased products [402] points to the need for the fast growth of industries producing environmentally friendly solutions and approaches, and the necessity for the industry to satisfy various end-user requirements at a competitive cost during their entire life cycle.

### 7.8. Consumer Perception and Acceptance

Several aspects such as safety, regulatory and economic considerations, consumers, and consumers’ perception of packaging influence the implementability and marketability of new packaging solutions. In recent years, consumers have become more aware of environmental issues caused by the inappropriate and uncontrolled landfilling of plastics, which can be a result of the lack of viable waste handling systems. This rising awareness led to the phenomenon of so-called “plastic bashing”. The trend and the growing demand of consumers to reduce plastics in their everyday lives, especially in packaging, have already resulted in resolutions banning multiple disposable plastics items, such as drinking straws and plastics bag. Consumers are looking for more sustainable solutions. However, as summarized by Otto et al. [403], consumer perceptions may differ from scientific facts. Consequently, assessing the perception, acceptance, and expectations of consumers towards new packaging solutions is important.

#### 7.8.1. Consumer Perception and Acceptance Regarding Biobased Food Packaging

The following subchapter will focus on consumers’ perception and acceptance of biobased packaging. Special focus will be given to biobased plastics. However, as data on the perception of biobased plastic packaging is limited, studies on environmentally friendly packaging and fibre-based packaging will also be considered.

The studies performed by Ottman (1998) [404] and Thøgersen (1999) [405] indicated that respondents regard environmentally friendly packaging as option when a) environmental impacts are recognized as considerable; and when b) no other relevant feature, such as price, quality, or the like, affects their decision about packaging. This means sustainability characteristics can determine purchase choice between two products or packed goods if both are otherwise perceived as equal.

When conducting a transnational study investigating “consumer attitudes towards biobased packaging” in France, Germany, and the United States of America, Herbes et al. [406] observed that consumers predominantly focus on the EoL attributes of packaging. This may seem logical considering that consumers interact most directly with packaging at this stage of life of packaging. However, the consumers in the different countries investigated differed in how they ranked the attributes “recyclability”, “reusability” and “biodegradability”. While in France respondents perceived packaging based on recyclable materials as the most environmentally friendly, German respondents prioritized packaging based on reusable materials.

Similarly, country-specific differences can also be observed regarding the consumer perception of different packaging types, as summarized in Table 1 [407]. Germany and Italy show very similar perception patterns, especially regarding the three top-rated and worst-rated packaging types. Respondents in both countries rate “glass bottles and jars” as the most sustainable packaging solution followed by “plastic films made from renewable, compostable raw material” as second and “paper-based cartons” as third. The worst-rated packaging type in both countries is “packaging combining plastic, paper, and aluminium foil” in tenth place with “aluminium foil wraps” in ninth place and “metal containers” in eighth place [407].

Interestingly, in France “glass bottles and jars” and “plastic films made from renewable, compostable raw material” are also ranked first and second, while “plastic bottles and containers that are fully recyclable” are ranked third [407]. The packaging type ranked second corresponds very well with that of another study, which reported that respondents in Germany as well as France only perceived plastic packaging made from renewable resources as more environmentally friendly if the plastics were also biodegradable [406]. Moreover, “packaging combining plastic, paper, and aluminium foil” and “aluminium foil wraps” were also ranked tenth and ninth, respectively, but in eighth place were “plastic bottles and containers made from recycled plastic materials”. Respondents in the United Kingdom showed the same patterns in their perceptions of the three worst-rated packaging types as France. Regarding the top three rated packaging types, some differences could be observed. Again, “glass bottles and jars” were rated as the most sustainable. In second place, however, were “paper-based cartons” followed by “flexible paper” [407].

Besides country-specific challenges, these findings indicate another more general problem. Consumers often harbour misconceptions. This is especially true regarding the differentiation of biodegradable and biobased plastics, the overestimation of biobased plastic’s biodegradability, and the underestimation of the recycling of biobased plastics [406,408,409,410].

Nonetheless, fibre-based packaging is more likely to be perceived correctly compared to other examples for bio-packaging. This means that consumers more often perceive fibre-based packaging, such as boards, as sustainable and environmentally friendly [403]. However, as demonstrated by Steenis et al. [411], this perception may depend on the specific packaging demonstrator. For instance, the mentioned study used tomato soup products varying in packaging design as stimuli. The authors found that a liquid carton was perceived more sustainable than a dry carton sachet design. However, upon evaluation by life cycle assessments, the dry carton sachet design was found to be more sustainable than the liquid carton design [411]. Nevertheless, the positive image that consumers have regarding fibre-based packaging is more likely to be backed by scientific evidence than the negative image that many consumers have of plastic in general.

The major challenge in assessing consumers’ perceptions relates to emotions being the key driver for certain purchase decisions [409]. This means that purchase intentions may or may not differ from purchase decisions, as purchase intentions may to some extent follow cognitive reasoning whereas purchase decisions are more spontaneous and a result of affective feelings. Consequently, the results of consumer perception studies based on online questionnaires may differ from the results of studies performed in person and with packaging samples [409,412].

Moreover, the study performed by Sijtsema et al. [409] observed that respondents were rather unfamiliar with “biobased” as a general concept and showed better responses when confronted with individual biobased products.

Taken together, it has been demonstrated that consumers tend to show positive perceptions of biobased packaging. However, they also harbour misconceptions and, therefore, need more guidance and more detailed information readily available to be able to correctly classify packaging in general.

#### 7.8.2. Consumer Perception and Acceptance Regarding the Use of SRM

Besides having an important role in the implementability and marketability of new packaging solutions, consumers have a significant influence on the circular economy strategy as their purchase decisions determine whether products are consumed in a circular manner or not. Consequently, it is important to assess consumer perception and acceptance of circular goods, i.e., products made from SRM [413]. As the literature dealing with consumer perception and acceptance of packaging made from SRM is very limited, studies analysing consumer perception and acceptance of recycled (fast-moving consumer) goods are used for extrapolation.

Several studies have indicated that consumers show higher purchase intentions towards SRM-derived fast-moving consumer goods, which also includes packaging, compared to textiles in terms of clothing [414,415]. More precisely: consumers express greater intentions to use a product made from recycled plastic when it is not touching the skin. Although these observations may be considered promising for personal care and transport packaging made from SRM, additional factors influence purchase intentions.

It is important to understand that consumer perception is at least partly if not mainly driven by the consumers’ experiences with and emotions towards certain materials. Consequently, the perceived sustainability and actual sustainability of a certain product or material may greatly differ, as demonstrated by Du Bois et al. [416]. In the mentioned study, for only three of ten samples, the perceived sustainability matched the corresponding actual sustainability. Four samples were overestimated and three samples were underestimated regarding their sustainability. The same study also found that recycled materials are perceived more sustainable if the texture is slightly rougher, the colour is less intense, the material is more matte and colourless rather than colourful [416]. These aspects are interesting and should be considered during product design to ensure commercial acceptance and success.

As demonstrated in recent studies, consumers’ purchase intentions towards recycled goods are significantly influenced by perceived image and perceived safety but not by perceived quality and environmental benefits [414,417]. This means that consumers are more likely to buy recycled goods if they have a positive image of these products in their mind and if they feel that these products are adequately safe. For packaging, circular design strategies are perceived more sustainable than linear design strategies, such as lightweighting packaging. Additionally, perceived naturalness and moral satisfaction also add to perceived sustainability, which influences consumers’ purchase intentions [418]. Among others, perceived naturalness is often associated with dull colours, nature photos and images, as well as logos that convey the impression of environmental friendliness [419,420]. These cues need to be taken into account and may also be relevant for biobased packaging.

In addition, consumers’ demographics should not be neglected. For instance, Russo et al. [421] found that age correlates to the willingness-to-pay. This means when comparing older and younger consumers with high green self-identities, the willingness-to-pay positively correlates with increasing consumers’ age. This is of special interest as recycled material is more expensive than virgin material as already stated in Section 6.2. In contrast, another study reported that whether or not recycled goods are perceived sustainable depends on the consumer’s awareness and knowledge regarding sustainability [414].

Taken together, consumers are likely to buy products contained in packaging made from SRM. However, certain aspects, such as packaging safety and image, may need further communication and explanation.

## 8. Conclusions

This review summarises the updated state of the art of the most recent advances in biobased polymers and coatings relevant for bioplastics, in particular PLA, PHA, bioPE and bioPET, and fibre-based packaging. The literature compilation indicates that there is a lot of ongoing research matched by an increasing market interest in developing new materials for biobased packaging as well as the recycling and upcycling of these materials.

In particular, PLA, PHA, and whey proteins are promising, well-studied polymers for biobased packaging films that have been applied and studied in multilayer films or in modified versions to further enhance barrier properties. Although the mentioned and other biobased materials were extensively studied as outlined in this review, more studies focusing on grafting technologies, biobased multilayers, and EoL options are still needed.

Moreover, the different recycling approaches and the potential for the application of post-consumer plastic derived from packaging structures was also reviewed. Special focus was given to consumer acceptance, marketability, future prospects, and challenges. In summary, the findings indicate a great potential of circular packaging designs with respect to more sustainability and circularity in the packaging industry.

## Figures and Tables

**Table 1:** Overview of the three top-rated and worst-rated packaging solutions adapted from Eriksson et al. [407] sorted by country.

	Rang of Corresponding Packaging Solution in			
Packaging Solution	France	Germany	Italy	United Kingdom
Paper-based cartons		3	3	2
Glass bottles and jars	1	1	1	1
Plastic films made from renewable, compostable raw materials	2	2	2	
Flexible paper				3
Plastic bottles and containers that are fully recyclable	3			
Metal containers		8	8	6
Plastic bottles and containers made from recycled materials	8			8
Aluminium foil wraps	9	9	9	9
Packaging combining plastic, paper, and aluminium foil	10	10	10	10

## Data Availability

No new data were created or analyzed in this study. Data sharing is not applicable to this article.
